# Clovamide, a Hydroxycinnamic Acid Amide, Is a Resistance Factor Against *Phytophthora* spp. in *Theobroma cacao*

**DOI:** 10.3389/fpls.2020.617520

**Published:** 2020-12-23

**Authors:** Benjamin J. Knollenberg, Guo-Xing Li, Joshua D. Lambert, Siela N. Maximova, Mark J. Guiltinan

**Affiliations:** ^1^ Plant Biology PhD Program ‐ Huck Institutes of the Life Sciences, Pennsylvania State University, University Park, PA, United States; ^2^ Department of Plant Sciences, Pennsylvania State University, University Park, PA, United States; ^3^ Department of Chemistry, Pennsylvania State University, University Park, PA, United States; ^4^ Department of Food Science, Pennsylvania State University, University Park, PA, United States; ^5^ Huck Institutes of the Life Sciences, Pennsylvania State University, University Park, PA, United States

**Keywords:** *Theobroma cacao*, *Phytophthora*, metabolomics, clovamide, hydroxycinnamic acid amide, black pod rot, polyphenol oxidase, oomycete

## Abstract

The hydroxycinnamic acid amides (HCAAs) are a diverse group of plant-specialized phenylpropanoid metabolites distributed widely in the plant kingdom and are known to be involved in tolerance to abiotic and biotic stress. The HCAA clovamide is reported in a small number of distantly related species. To explore the contribution of specialized metabolites to disease resistance in cacao (*Theobroma cacao* L., chocolate tree), we performed untargeted metabolomics using liquid chromatography – tandem mass spectrometry (LC-MS/MS) and compared the basal metabolite profiles in leaves of two cacao genotypes with contrasting levels of susceptibility to *Phytophthora* spp. Leaves of the tolerant genotype ‘Scavina 6’ (‘Sca6’) were found to accumulate dramatically higher levels of clovamide and several other HCAAs compared to the susceptible ‘Imperial College Selection 1’ (‘ICS1’). Clovamide was the most abundant metabolite in ‘Sca6’ leaf extracts based on MS signal, and was up to 58-fold higher in ‘Sca6’ than in ‘ICS1’. *In vitro* assays demonstrated that clovamide inhibits growth of three pathogens of cacao in the genus *Phytophthora*, is a substrate for cacao polyphenol oxidase, and is a contributor to enzymatic browning. Furthermore, clovamide inhibited proteinase and pectinase *in vitro*, activities associated with defense in plant-pathogen interactions. Fruit epidermal peels from both genotypes contained substantial amounts of clovamide, but two sulfated HCAAs were present at high abundance exclusively in ‘Sca6’ suggesting a potential functional role of these compounds. The potential to breed cacao with increased HCAAs for improved agricultural performance is discussed.

## Introduction

The Food and Agriculture Organization of the United Nations has declared 2020 as the International Year of Plant Health, one of the major goals of which being to “keep plants healthy while protecting the environment” (FAO, 2019). This is no small challenge since an estimated 17–30% of major crops are lost yearly to disease (Savary et al., 2019), and this crop loss has been met with ever-increasing use of chemical pesticides associated with negative environmental impacts (Maggi et al., 2019; Sharma et al., 2019). Increasing host plant resistance to pathogens by traditional breeding or genetic engineering is one important strategy to reduce crop losses and the use of pesticides. This approach necessitates a more complete understanding of plant resistance, which utilizes an astonishing array of interconnected mechanisms (Wang et al., 2019), including production of defense-related small molecules, or metabolites, with a wide range of structures and activities. In this work we describe clovamide, a hydroxycinnamic acid amide (HCAA) metabolite, as an important resistance factor in cacao (*Theobroma cacao*, chocolate) against pathogens in the genus *Phytophthora*, an oomycete (water mold) genus comprised of over 100 species, many of which are globally important plant pathogens with broad host ranges (Thines, 2014).

Plant secondary or specialized metabolites are small molecules (phenolics, terpenoids, alkaloids, etc.) well known for their role in abiotic and biotic stress tolerance (Dixon, 2001; Hartmann, 2007; Isah, 2019). HCAAs, also referred to as *N*-phenylpropenoyl-*L*-amino acids (NPAs; Lechtenberg et al., 2012), are a class of specialized metabolites that play a significant role in plant stress tolerance, particularly in defense against necrotrophic and hemi-biotrophic pathogens (Martin-Tanguy, 1985; Macoy et al., 2015). HCAAs typically form *via* the amide condensation of hydroxycinnamoyl-CoA thioesters and amines (Bontpart et al., 2015; Petersen, 2016). They contribute to stress tolerance in numerous plant species due to their high antioxidant activity (Zacares et al., 2007), cell wall reinforcing properties (Gunnaiah et al., 2012), or direct antimicrobial activity (Newman et al., 2001; Kyselka et al., 2018). The role of HCAAs in defense is supported by *in vivo* evidence. An *Arabidopsis* mutant of *AtACT*, which catalyzes formation of agmatine‐ and putrescine-containing HCAAs, was more susceptible to the necrotrophic fungus *Alternaria brassicola* (Muroi et al., 2009). Furthermore, transgenic *Torenia hybrida* overexpressing *AtACT* exhibited enhanced resistance to the necrotrophic fungus *Botrytis cinerea* (Muroi et al., 2012). Additionally, potato overexpressing *AtACT* and a MATE transporter required for excretion of coumaroyl-agmatine into the extracellular space had enhanced resistance to *Phytophthora infestans* (Dobritzsch et al., 2016). Tomato overexpressing *hydroxycinnamoyl-CoA: tyramine-N-hydroxycinnamoyl transferase* (*SlTHT*) had elevated levels of the HCAAs coumaroyl‐ and feruloyl-tyramine and enhanced resistance to *Pseudomonas syringae* (Campos et al., 2014). High relative accumulation of HCAAs in potato in response to *P. infestans* infection is associated with tolerance to the pathogen (Yogendra and Kushalappa, 2016). To our knowledge, the role of HCAAs in cacao resistance to *Phytophthora* spp. has not been described to date.

Clovamide, an HCAA consisting of caffeic acid and *L*-3, 4-dihydroxyphenylalanine (*L*-DOPA) moieties joined by an amide bond, is reported in a small number of distantly related plant species, including *Trifolium* spp. (Yoshihara et al., 1977; Kolodziejczyk-Czepas et al., 2017), *Vernonia fastigiata* (Masike et al., 2017), *Dalbergia* spp. (Van Heerden et al., 1980; El-Sharawy et al., 2017), *Acmella oleracea* (Nascimento et al., 2020), and cacao (Sanbongi et al., 1998). Clovamide has garnered considerable attention for its potential human health benefits as an anti-inflammatory and neuroprotective compound (Park et al., 2007, 2017; Fallarini et al., 2009; Zeng et al., 2011; Kolodziejczyk-Czepas et al., 2017; Tsunoda et al., 2018) and as an anti-microbial with activity against the human pathogens influenza A subtype H5N1 (El-Sharawy et al., 2017), *Trypanosoma evansi* (same source), and *Heliobacter pylori* (Niehues et al., 2011). Clovamide’s role in plant defense has not been described to our knowledge, although treatment of red clover seedlings with the plant defense hormone jasmonic acid induced clovamide accumulation in roots, suggesting a role in defense (Tebayashi et al., 2000).

Cultivation of cacao provides the raw material for a multi-billion dollar international chocolate industry and is an important source of income to over 5 million farmers worldwide (Ingram, 2015; Beg et al., 2017). Pathogen pressure limits cacao production by up to 30% annually and pathogens in the genus *Phytophthora* (*P. megakarya, P. palmivora, P. tropicalis, P. citropthora, P. heveae*, etc.), which cause Black pod rot (BPR), make the greatest contribution to this loss (Ploetz, 2007).

Various management strategies for BPR exist but have not addressed the crop losses in a satisfactory way. Partial control of BPR can be achieved in the field with chemical and cultural strategies, such as pruning, sanitation, more frequent harvesting of ripe pods or fruits (Guest, 2007). These control strategies, however, are labor and knowledge intensive and their adoption is often hindered by limited access to extension services (Hebbar, 2007) or the high cost of inputs (OpokuI et al., 2007; Wessel and Quist-Wessel, 2015). With these limitations in mind, genetic improvement of cacao for BPR resistance is widely considered as the most promising strategy to combat the disease at scale (Flament et al., 2001; Lanaud et al., 2009; Thevenin et al., 2012). Understanding the biological mechanisms of cacao resistance to BPR would help breeders to more quickly incorporate resistance traits into high yielding varieties.

Several studies have been undertaken to elucidate cacao defense mechanisms against BPR. Since the first genetic map of cacao in 1995 (Lanaud et al., 1995), several quantitative trait loci (QTL) analyses have identified over 60 QTL associated with BPR resistance (Lanaud et al., 2009; Akaza et al., 2016; Barreto et al., 2018), highlighting the oligogenic nature of BPR resistance and the challenge this presents to breeders. Functional characterization of genes and associated mechanisms underlying QTL has not been demonstrated. Since the advent of next-generation sequencing technologies and the sequencing of the first cacao genome (Argout et al., 2011) large scale gene expression, or transcriptomics, studies have also been used to study BPR resistance. Such studies have revealed several defense mechanisms potentially important for distinguishing tolerant from susceptible genotypes, including higher relative superoxide production in response to salicylic acid treatment (Fister et al., 2015), more rapid transcriptional response to infection (Pokou et al., 2019), and higher relative infection-induced expression of genes putatively involved in hormone signaling and protease inhibition in tolerant genotypes (Legavre et al., 2015).

Here, we present the results of an untargeted liquid chromatography–tandem mass spectrometry (LC-MS/MS) metabolomics analysis comparing leaf metabolites between two well-known cacao genotypes contrasting for resistance to pathogens in the genus *Phytophthora*: the tolerant ‘Scavina 6’ (‘Sca6’) and the susceptible ‘Imperial College Selection 1’ (‘ICS1’) (Lanaud et al., 2009; Fister et al., 2015, 2019). We observed that ‘Sca6’ leaves accumulated much higher levels of several HCAAs relative to ‘ICS1.’ The most abundant metabolite feature in ‘Sca6’ extracts was clovamide, which was up to ~58-fold higher in ‘Sca6’ leaves based on targeted quantification. Pure synthetic clovamide inhibited *in vitro* growth of *P. megakarya, P. palmivora*, and *P. tropicalis*, three major causal agents of BPR, at a physiologically relevant concentration. Additionally, clovamide inhibited proteolysis and pectolysis *in vitro*, was confirmed as a substrate for cacao polyphenol oxidase, and contributed to enzymatic browning in tissue damage assays. Both genotypes tested accumulated clovamide in fruit epidermal peels, but two sulfated HCAAs were accumulated at high levels exclusively in ‘Sca6.’ Taken together, these results indicate that clovamide accumulation is an important factor in ‘Sca6’ tolerance to *Phytophthora* spp. in leaves and sulfated HCAAs may play a similar role in fruit peels.

## Results

### Metabolomics Reveals High Accumulation of HCAAs in Leaves of ‘Sca6’ Relative to ‘ICS1’

**Figure 1:**
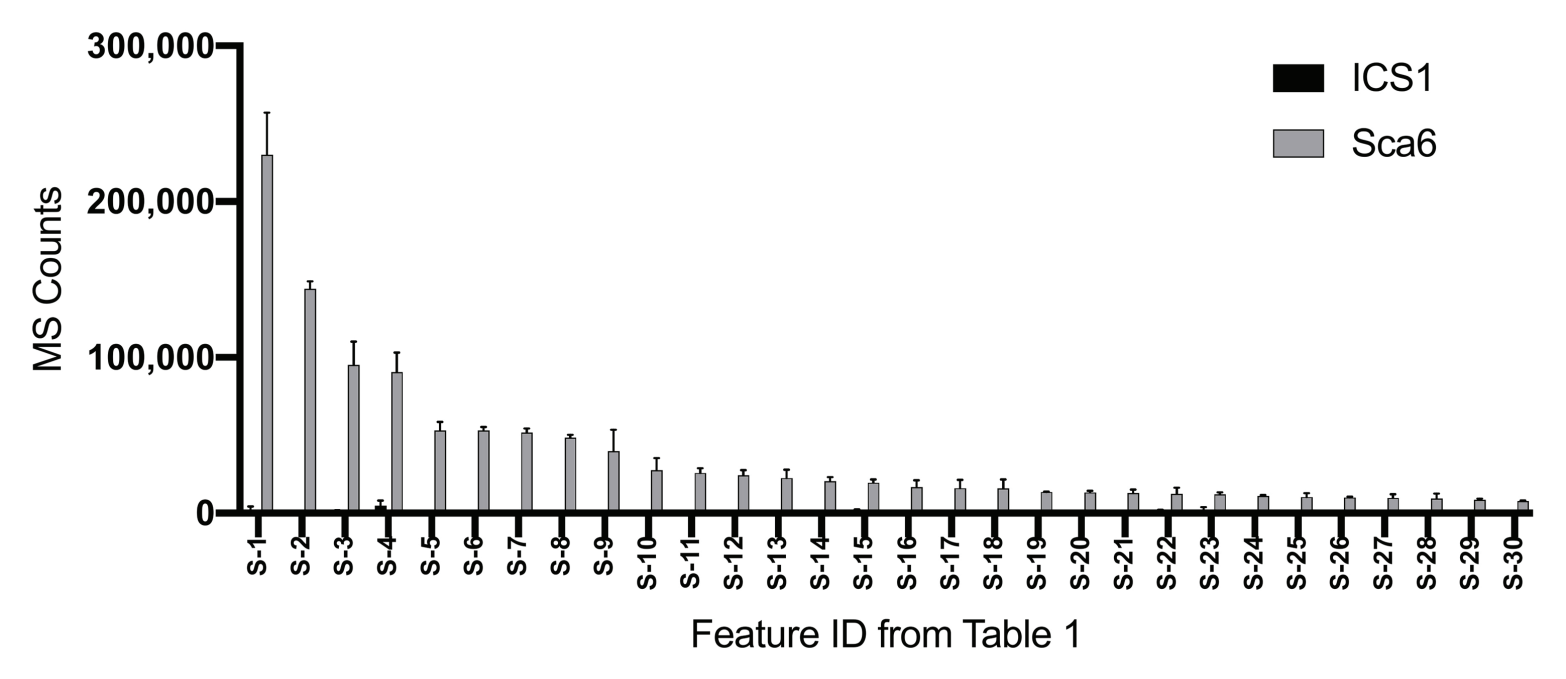
Relative abundance of the 30 most abundant features in ‘Sca6’ of those >5-fold higher than in ‘ICS1’ (*p* < 0.05). Feature IDs (S-1 through S-30) match those in Table 1. MS counts represent mass spectrometer signal intensity of peaks integrated in XCMS Online (Tautenhahn et al., 2012). Error bars represent standard deviation. *n* = 3.

Untargeted LC-MS/MS metabolomics was performed to compare basal (not infected) metabolite profiles in methanolic extracts of intermediate developmental “stage C” leaves (Mejía et al., 2012) from the tolerant ‘Sca6’ and the susceptible ‘ICS1’ cacao genotypes to generate a dataset with 1,719 metabolite features (signals of a particular mass to charge ratio, “m/z”, and retention time). Three statistical filters were applied to prioritize metabolite features for annotation. Firstly, features were selected that were significantly different between genotypes (*p* < 0.05). There were 364 features higher in ‘Sca6’ and 371 higher in ‘ICS1’ (*p* < 0.05). Secondly, features with a fold-difference of 5 or higher were selected, resulting in 183 features higher in ‘Sca6’ and 121 higher in ‘ICS1.’ Thirdly, of those higher in ‘Sca6’ (>5-fold, *p* < 0.05), the 30 most abundant features in ‘Sca6’ were selected for structural annotation. Mass spectrometer signal intensities for these top 30 features (S-1 through S-30) are presented in Figure 1 with their corresponding putative annotations in Table 1. A full list of MS/MS fragments for each metabolite feature is provided in Supplementary Data File 1.

**Table 1:** Putative annotations of the 30 most abundant features in ‘Sca6’ of those >5-fold higher than in ‘ICS1’ (*p* < 0.05).

Feature ID in Figure 1	M/Z	RT (min)	Top 4 MS/MS fragments	Annotation	Fold up in ‘Sca6’
S-1	717.197	5.64	222.04, 178.05, 358.09, 161.02	Adduct/isotope of S-4 (Caffeoyl-DOPA)	95.1
S-2	685.207	6.25	178.05, 222.04, 145.03, 342.10	Adduct/isotope of S-8 (Coumaroyl-DOPA)	2636.4
S-3	718.200	5.65	NA	Adduct/isotope of S-4 (Caffeoyl-DOPA)	98.7
**S-4**	**358.094**	**5.65**	**135.04, 161.02, 178.05, 160.04**	**Caffeoyl-DOPA/Clovamide [M-H]** ^**-**^	**18.8**
S-5	715.181	5.65	715.18, 553.14, 312.09, 286.11	Adduct/isotope of S-4 (Caffeoyl-DOPA)	69.7
S-6	686.211	6.25	NA	Adduct/isotope of S-8 (Coumaroyl-DOPA)	2705.4
**S-7**	**326.105**	**6.94**	**119.05, 145.03, 134.06, 146.06**	**Coumaroyl-Tyrosine [M-H]** ^**−**^	**63.5**
**S-8**	**342.099**	**6.27**	**135.04, 119.05, 161.02, 163.04**	**Coumaroyl-DOPA [M-H]** ^**−**^	**82.5**
S-9	699.187	6.29	356.08, 206.04,…, 342.10 (7th)	Adduct/isotope of S-8 (Coumaroyl-DOPA)	2221.6
**S-10**	**386.090**	**6.96**	**224.06, 137.02, 232.02, 135.05**	**Sinapoyl-Tyrosine [M-H]** ^**−**^	**192.5**
S-11	716.186	5.65	NA	Adduct/isotope of S-4 (Caffeoyl-DOPA)	67.8
S-12	719.203	5.65	NA	Adduct/isotope of S-4 (Caffeoyl-DOPA)	59.9
**S-13**	**365.116**	**8.50**	**135.05, 142.07, 161.02, 229.06**	**Caffeoyl-Tryptophan [M-H]** ^**−**^	**825.8**
S-14	359.097	5.64	NA	Adduct/isotope of S-4 (Caffeoyl-DOPA)	20.0
**S-15**	**456.062**	**5.68**	**96.96, 79.96,…, 358.10 (5th)**	**Caffeoyl-DOPA, Alkyl-Sulfated [M-H]** ^**−**^	**13.3**
**S-16**	**358.094**	**5.38**	**135.05, 161.02, 178.05, 133.03**	**Caffeoyl-DOPA/Clovamide [M-H]** ^**−**^	**62.1**
S-17	700.190	6.29	NA	Adduct/isotope of S-8 (Coumaroyl-DOPA)	1962.5
S-18	356.079	6.21	137.02, 160.04, 125.02, 218.04	Unknown	132.6
S-19	687.213	6.25	NA	Adduct/isotope of S-8 (Coumaroyl-DOPA)	1049.1
S-20	683.192	6.27	296.09, 145.03,…, 342.10 (7th)	Adduct/isotope of S-8 (Coumaroyl-DOPA)	337.4
**S-21**	**372.110**	**7.01**	**135.04, 146.04, 218.05, 250.07**	**Feruloyl-DOPA [M-H]** ^**−**^	**22.0**
S-22	711.363	13.30	223.13, 665.35, 459.22, 2015.12	Unknown	9.1
S-23	797.437	13.62	751.42, 223.13, 309.21, 797.43	Unknown	5.1
S-24	327.108	6.94	NA	Adduct/isotope of S-7 (Coumaroyl-Tyrosine)	62.6
**S-25**	**452.067**	**7.18**	**452.07, 178.05,…, 372.11 (6th)**	**Feruloyl-DOPA, Aryl-Sulfated [M-H]** ^**−**^	**393.8**
S-26	343.103	6.27	NA	Adduct/isotope of S-8 (Coumaroyl-DOPA)	78.9
**S-27**	**326.105**	**8.61**	**135.05, 161.02, 147.04, 133.03**	**Caffeoyl-Phenylalanine [M-H]** ^**−**^	**557.9**
S-28	388.105	6.12	137.02, 135.04, 125.02, 209.05	Unknown	333.3
**S-29**	**440.067**	**6.27**	**96.96, 79.96,…342.10 (9th)**	**Caffeoyl-Tyrosine, Alkyl-Sulfated [M-H]** ^**−**^	**90.5**
S-30	684.196	6.25	NA	Adduct/isotope of S-8 (Coumaroyl-DOPA)	147.3

Feature IDs (S-1 through S-30) match those in Figure 1. “m/z” is median mass/charge ratio. “RT” is median retention time in minutes. The four most intense MS/MS fragments are reported, and in some cases less intense signals that were diagnostic are presented with their rank in abundance relative to other MS/MS signals in parentheses. Fold changes (‘Sca6’ vs. ‘ICS1’) were calculated in XCMS Online (Tautenhahn et al., 2012). Suspected molecular ions ([M-H]^−^) are bold.

Of the top 30 features higher in ‘Sca6’ (>5-fold, *p* < 0.05), 26 were annotated as hydroxycinnamic acid amides (HCAAs), including molecular ions ([M-H]^−^) of caffeoyl-DOPA/clovamide (S-4, S-16), coumaroyl-tyrosine (S-7), coumaroyl-DOPA (S-8), sinapoyl-tyrosine (S-10), caffeoyl-tryptophan (S-13), and feruloyl-DOPA (S-21) (Table 1). Several features were putatively annotated as adducts or stable isotopes of their respective molecular ions based on shared retention time with a mass that is a multiple of, and/or a fragment in MS/MS that matches the associated molecular ion. For example, S-1 has a mass consistent with a coupling of two molecules of caffeoyl-DOPA/clovamide ([2 M-H]^−^) shares a retention time with S-4 (caffeoyl-DOPA/clovamide), has a fragment in MS/MS matching that of S-4, and is therefore a likely adduct of S-4. Metabolite feature S-3 is likely a stable isotope of S-1 ([M + 1-H]^−^), although for simplicity distinction between adducts and stable isotopes is not made in Table 1.

**Figure 2:**
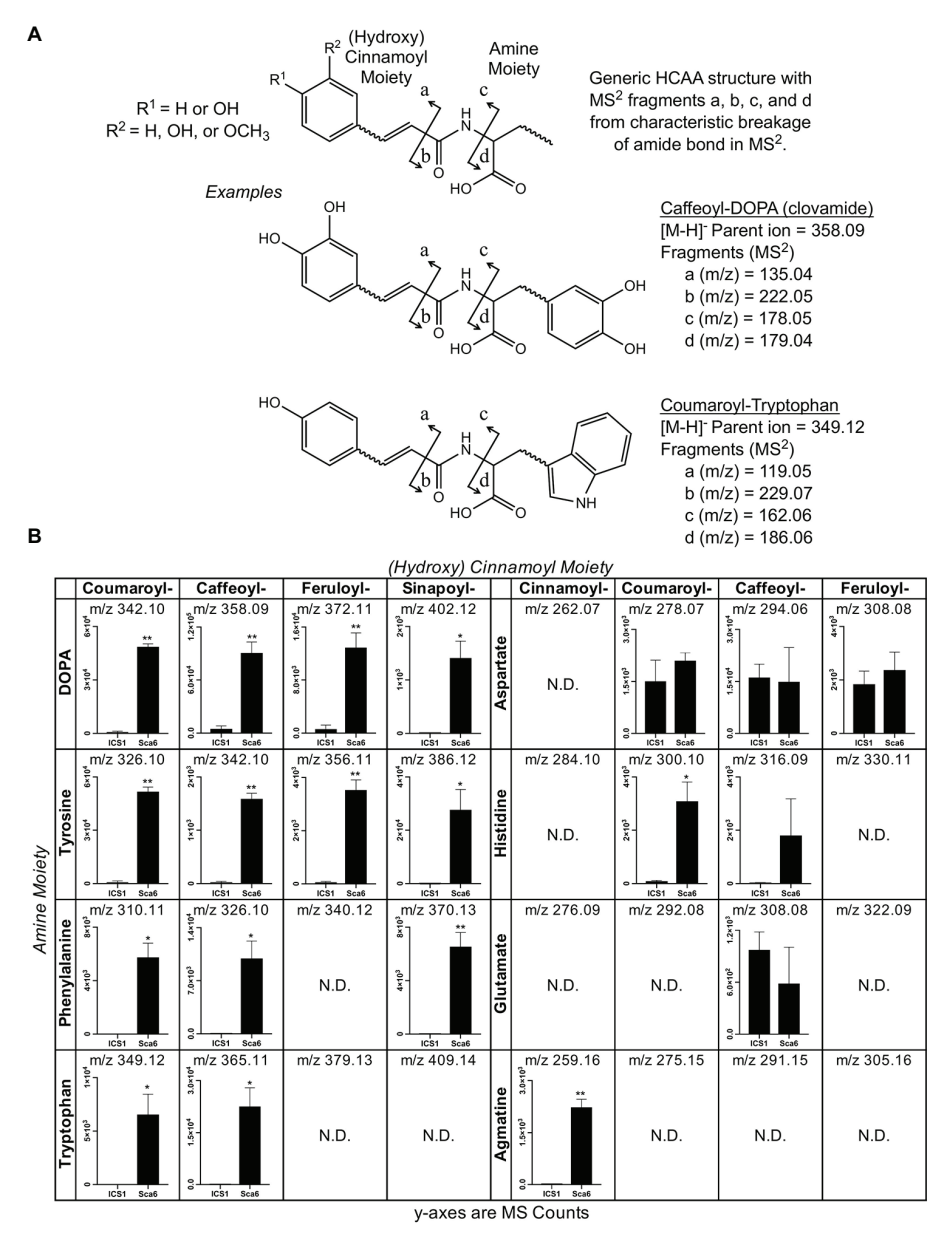
Semi-targeted LC-MS/MS analysis of HCAAs in leaf tissue (‘ICS1’ and ‘Sca6’). **(A)** Generic HCAA structure and diagnostic MS/MS fragments based on clovamide fragmentation (Arlorio et al., 2008) used to predict HCAAs in LC-MS/MS data. Two examples, Caffeoyl-DOPA (clovamide) and Coumaroyl-Tryptophan, are shown. **(B)** Relative abundance of putative HCAAs detected in LC–MS data. “N.D.” = not detected. ^*^ *p* < 0.05, ^**^ *p* < 0.01 (Welch’s *t*-test), *n* = 3. Error bars represent standard deviation.

Since most of the 30 metabolite features in Table 1 were in the class of HCAAs, a semi-targeted search for other HCAAs was performed by generating a library of hypothetical HCAA MS^1^ parent ion masses ([M-H]^−^) and MS/MS fragment masses for 250 compounds consisting of a (hydroxy) cinnamic acid moiety and an amine moiety (see methods). Examples of diagnostic HCAA fragments are shown in Figure 2A. Full MS/MS spectra and fold changes between genotypes for all predicted HCAAs in the dataset are available in Supplementary Data File 2.

Twenty-five metabolite features ([M-H]^−^) in the LC-MS/MS data were putatively annotated as HCAAs using this predictive approach. Some predicted HCAAs had more than one metabolite feature match. For example, feruloyl-DOPA had two matches with retention times ~22 s apart, which may represent *cis/trans* isomers at the double bond in the ferulic acid moiety, although such isomers cannot be distinguished by LC-MS/MS. For HCAAs with multiple matches in the data set, MS signal intensity for the most abundant is presented (Figure 2B). HCAAs containing amine moieties with aromatic side chains were all significantly higher in ‘Sca6’ than ‘ICS1’. HCAAs containing aspartate or glutamate amine moieties were produced at the same amount in both genotypes. Coumaroyl-histidine and cinnamoyl-agmatine were higher in ‘Sca6’, while caffeoyl-histidine appeared higher but was not statistically significant (*p* ≈ 0.155).

Clovamide (caffeoyl-DOPA) was the most abundant signal in ‘Sca6’ extracts based on LC-MS signal and was ~41.5-fold higher than in ‘ICS1’ based on combined signal intensities of the molecular ion and associated adducts and stable isotopes (S-1, S-3:S-5, S-11, S-12, and S-14 from Table 1). Based on these results, previous reports of clovamide as a polyphenol oxidase (PPO) substrate and proteolysis inhibitor (Sullivan and Hatfield, 2006; Sullivan and Zeller, 2013), and previous work citing PPO-mediated browning as a major factor in BPR resistance of ‘Sca6’ (Spence, 1961), clovamide was selected for organic synthesis, targeted quantification, and further functional characterization to investigate its role in resistance to *Phytophthora* spp.

### Clovamide Synthesis

In order to obtain sufficient amounts of pure clovamide for functional testing, organic synthesis was performed. Clovamide, (−)-N-[3',4'-dihydroxy-(E)-cinnamoyl]-3-hydroxy-L-tyrosine, was synthesized from *trans*-caffeic acid and L-DOPA methyl ester following methods by Xie et al. (2013). Structural confirmation was done by ^1^H NMR to obtain the following spectra, consistent with previous reports (Stark and Hofmann, 2005):

^1^H NMR (500 MHz, DMSO-d_6_) δ 12.59 (s, 1H), 9.37 (s, 1H), 9.13 (s, 1H), 8.74 (s, 1H), 8.69 (s, 1H), 8.20 (d, *J* = 8.0 Hz, 1H), 7.19 (d, *J* = 15.7 Hz, 1H), 6.93 (d, *J* = 2.0 Hz, 1H), 6.82 (dd, *J* = 8.2, 2.0 Hz, 1H), 6.73 (d, *J* = 8.1 Hz, 1H), 6.62–6.59 (m, 2H), 6.47 (dd, *J* = 8.1, 2.0 Hz, 1H), 6.40 (d, *J* = 15.7 Hz, 1H), 4.45–4.41 (m, 1H), 2.89 (dd, *J* = 13.9, 4.9 Hz, 1H), 2.72 (dd, J = 13.9, 9.2 Hz, 1H).

### Clovamide Is Higher in ‘Sca6’ Than in ‘ICS1’ Throughout Leaf Development, but Not in Fruit Peels

Due to the apparently high degree of clovamide adduct formation in LC-MS (Table 1) and the potential for matrix effects interfering with quantification by LC-MS (Taylor, 2005), High-performance liquid chromatography coupled with a diode array detector (HPLC-DAD) was chosen as the method for quantification of clovamide. Five serial dilutions (1:5) of clovamide were prepared from 0.05 to 0.00008 mg/ml and analyzed by HPLC-DAD to generate a calibration curve (*r* ^2^ = 0.9993; limit of detection = 1.845 μg/ml; limit of quantification = 6.149 μg/ml).

**Figure 3:**
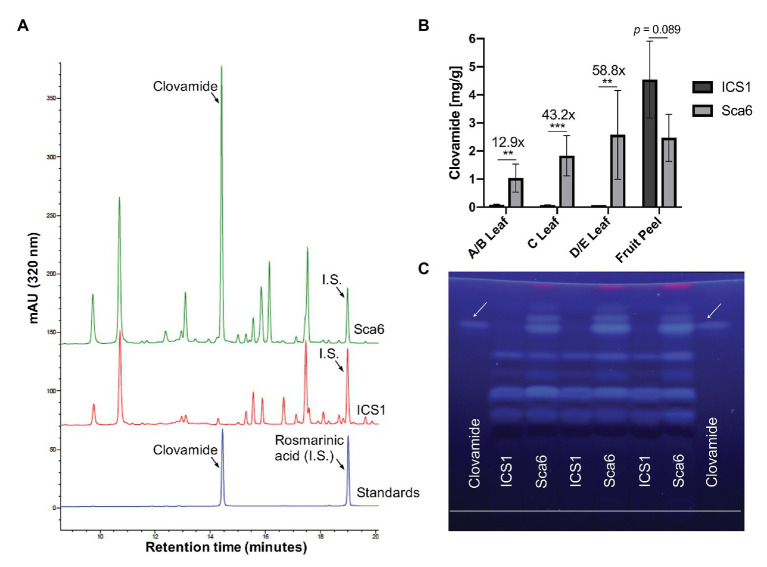
Clovamide detection and quantification in cacao leaf and fruit peel. **(A)** High performance liquid chromatography – diode array detector (HPLC-DAD, 320 nm) chromatogram of ‘Sca6’ and ‘ICS1’ stage C leaf extracts, including UV absorbance spectra for peaks of interest. I.S. = internal standard. **(B)** Clovamide content (mg/gram tissue) from HPLC-DAD. ^**^ *p* < 0.01, ^***^ *p* < 0.001, *t*-test (*n* = 5 for leaves, *n* = 3 for fruit peel). Error bars represent standard deviation. **(C)** Thin-layer Chromatography (TLC) plate with clovamide standard and stage C leaf extracts from ‘ICS1’ and ‘Sca6’. White line drawn near bottom of plate is origin of sample loading. Photograph taken under 365 nm UV excitation.

Three leaf developmental stages as defined by Mejía et al. (2012) and fruit peel (exocarp and ~2 mm mesocarp) from one fruit pod developmental stage were analyzed for clovamide content. Extracts from stage A/B leaf (young and red), stage C leaf (intermediate and bronze), stage D/E leaf (mature and green), and fruit peel (mature, unripe, and 4–5 months old) tissues were analyzed using HPLC-DAD and clovamide was quantified (Figures 3A,B). In stage A/B, C, and D/E stage leaves clovamide was 12.9-, 43.2-, and 58.8-fold higher, respectively, in ‘Sca6’ than ‘ICS1’ (*p* < 0.01). In contrast to leaves, both genotypes produced substantial amounts of clovamide in pod peels and ‘ICS1’ had ~1.8-fold more than ‘Sca6’ (*p* = 0.089).

Extracts from stage C leaves were also analyzed using thin-layer chromatography (TLC; Figure 3C). The synthetic standard fluoresced blue under long wave ultraviolet excitation (365 nm), had a retention factor of 0.81 ± 0.004, and aligned with a bright band in ‘Sca6’ extracts but not in ‘ICS1.’

### Clovamide Participates in Polyphenol Oxidase-Mediated Enzymatic Browning

**Figure 4:**
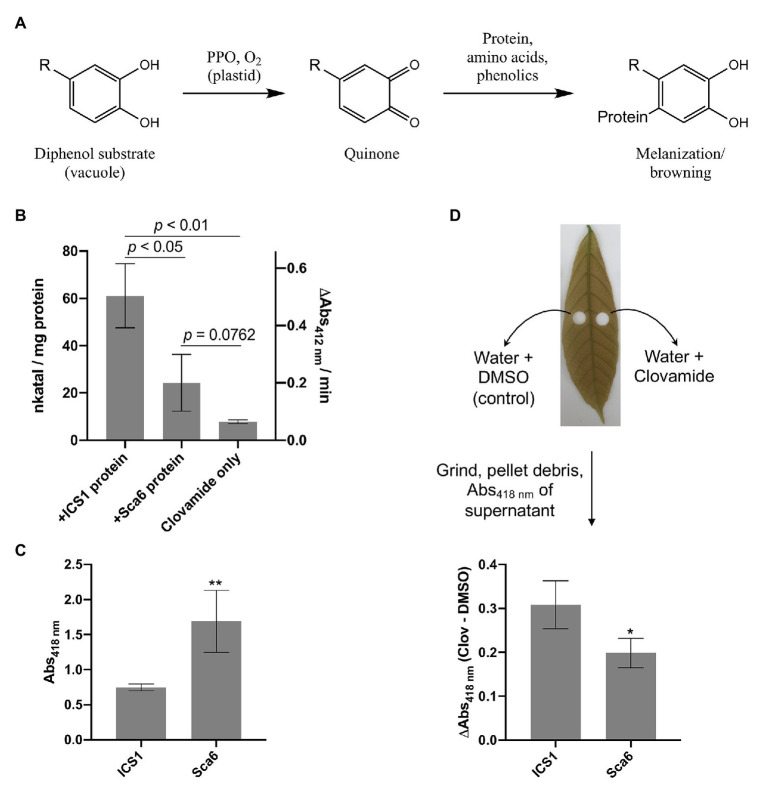
Clovamide’s contribution to enzymatic browning as a polyphenol oxidase (PPO) substrate. **(A)** Diagram of PPO-mediated formation of quinones from generic *o*-diphenol substrate and subsequent melanization/browning. Adapted from Yamane et al. (2010). **(B)** PPO activity (quinone formation) of ‘ICS1’ and ‘Sca6’ stage C leaf protein extracts with clovamide as substrate. [Clovamide] = 0.5 mM in all treatments shown. *p*-values from *t*-test, *n* = 3. **(C)** Browning in supernatant (Abs_418 nm_) of ground stage C leaf disks in water (^**^ *p* < 0.01, *n* = 4). **(D)** Enhanced browning in supernatant (ΔAbs_418 nm_) by addition of clovamide (~27.7 μg clovamide added per leaf disk, see methods; ^*^ *p* < 0.05, *n* = 4). Clov = clovamide. Error bars in **(B–D)** represent standard deviation.

Work by Sullivan and Zeller (2013) has demonstrated that clovamide can inhibit proteolysis in forage crops (~50%), likely as a result of its oxidation and crosslinking with proteins, and that this effect was enhanced by PPO (~80%). This system of quinone generation by PPO-mediated oxidation of phenolics and subsequent crosslinking with proteins is a process known as enzymatic browning or melanization (Yamane et al., 2010; Figure 4A).

To determine if clovamide is a substrate of cacao PPO, total protein extracts from stage C leaf were assayed for PPO activity with clovamide as a substrate (Figure 4B). Leaf protein extracts of ‘ICS1’ had ~2.5-fold higher PPO activity than ‘Sca6’ (*p* < 0.05). The PPO activity detection method employed was a TNB quinone trap (Esterbauer et al., 1977), which can detect non-enzymatic oxidation of *o*-diphenols to quinones as well as PPO-mediated oxidation. Clovamide alone showed detectable quinone formation, indicating non-enzymatic oxidation. Quinone formation was enhanced by the presence of ‘Sca6’ (~3.1-fold, *p* = 0.0762) and ‘ICS1’ (~7.8-fold, *p* = 0.0024) protein extracts compared to clovamide alone, indicating PPO activity towards clovamide.

Further PPO activity assays were performed with additives to test for latent PPO activity (SDS), peroxidase interference in quinone formation (catalase), and PPO inhibition (kojic acid). Sodium dodecyl sulfate (SDS) has been reported to enhance or activate latent activity of PPO from some plant species for certain phenolic substrates (Moore and Flurkey, 1990; Jiménez and García-Carmona, 1996; Gandía-Herrero et al., 2005; Winters et al., 2008; Derardja et al., 2017). Inclusion of SDS at 0.25% (w/v) in this work did not enhance quinone formation, indicating that cacao leaf PPO does not display latent activity towards clovamide (Supplementary Figure 1). Inclusion of catalase did not affect PPO activity, while a significant reduction in PPO activity was measured for ‘ICS1’ protein extracts in the presence of 5 mM kojic acid (Supplementary Figure 1). Catalase eliminates peroxide formed by peroxidases, which can confound PPO measurements by contributing to quinone formation (Gertzen and Escobar, 2014), and kojic acid is a known PPO inhibitor (Chen et al., 1991). Taken together, these results show that the enhanced quinone formation in the presence of cacao protein extracts is likely due to PPO.

Since clovamide was more abundant in ‘Sca6’ but PPO activity was higher in ‘ICS1,’ a browning assay was developed to measure the extent of browning in ground stage C leaf tissue (Supplementary Figure 2 for assay development). Leaf disks were ground in water and browning/melanization (Abs_418 nm_) was measured in the supernatant after a 30-min incubation period. This provides a combined readout of PPO activity and phenolic substrate availability by measuring the final product, that is browning or melanization. Supernatant from ground ‘Sca6’ tissue produced ~2.26-fold more browning than ‘ICS1’ (*p* < 0.01; Figure 4C). This demonstrates that even though ‘ICS1’ leaves had higher PPO activity, PPO substrate availability determines the extent of browning.

Clovamide was added to the leaf disk browning assays to test for enhancement of browning (Figure 4D). Clovamide addition enhanced browning (Abs_418 nm_) relative to the solvent control in ‘Sca6’ by 0.20 ± 0.034 and in ‘ICS1’ by 0.31 ± 0.055 (both *p* < 0.01, paired *t*-test). The contribution to absorbance of clovamide in the absence of leaf tissue (Abs_418 nm_ = 0.016) does not account for the observed enhancement in browning, so the browning enhancement was dependent on oxidation by leaf proteins. Clovamide addition caused a greater enhancement of browning (ΔAbs_418 nm_) in ‘ICS1’ than ‘Sca6’ (*p* < 0.05, Figure 4D), likely due to the higher PPO activity in ‘ICS1’ leaves (Figure 4B).

### Clovamide Inhibits Proteinase K and Pectinase

Sullivan and Zeller (2013) reported clovamide as a potent inhibitor of proteolysis in forage, an effect that was enhanced by PPO activity. Protease inhibitors have been demonstrated as important resistance (and virulence) factors in plant-pathogen interactions (Jashni et al., 2015), which suggests clovamide may contribute to BPR resistance in this capacity. To explore this further, a proteolysis assay was performed to test if cacao leaf extracts can enhance proteolysis inhibition of clovamide.

**Figure 5:**
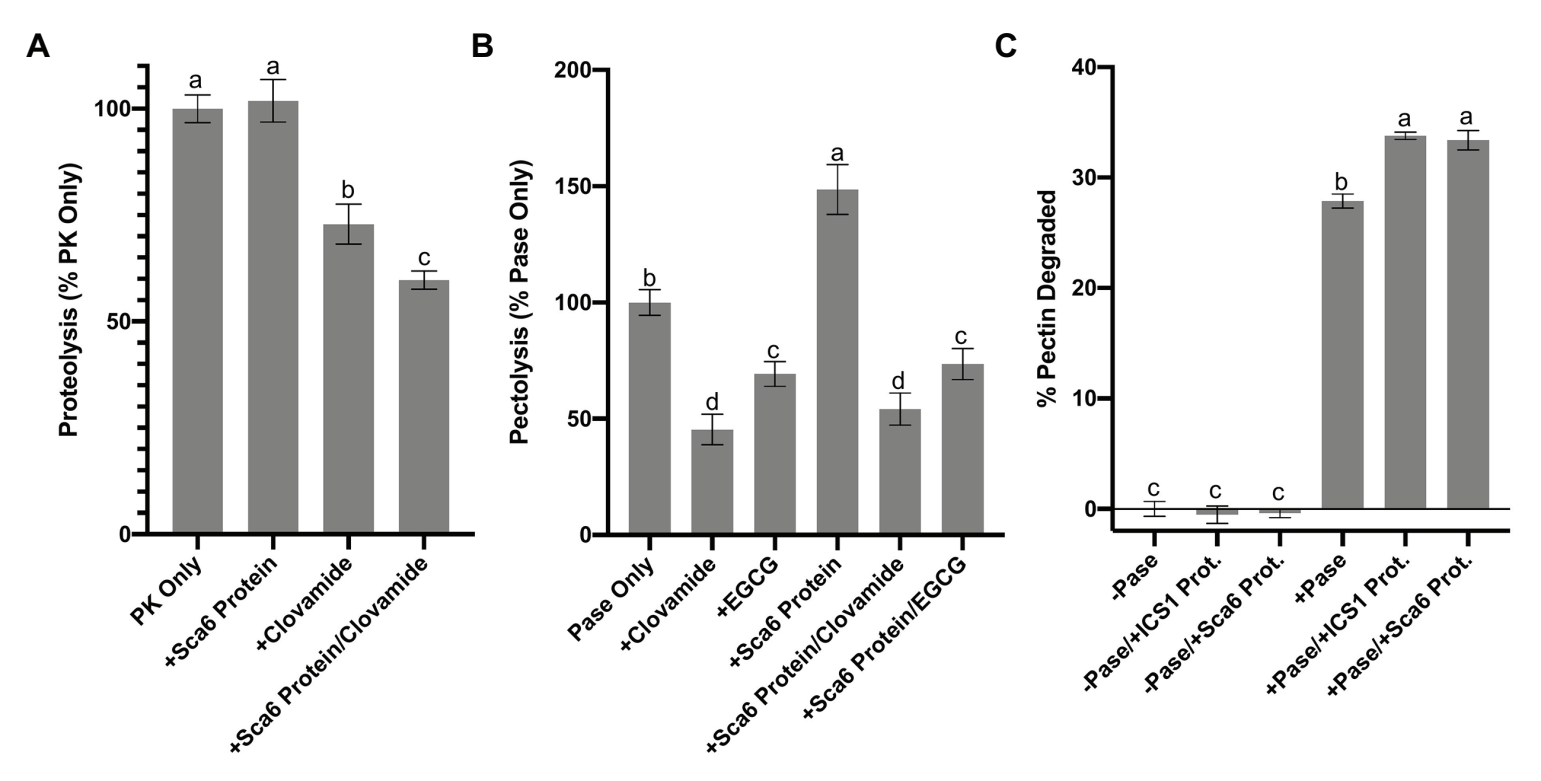
Enzyme inhibition by clovamide and effect of cacao stage C leaf protein pectinase activity. **(A)** Proteolysis inhibiton by clovamide. “PK” = proteinase K. PK included in all treatments shown, with the addition of ‘Sca6’ protein, clovamide (2 mM), or both. Data represents two experiments (*n* = 5 from each). **(B)** Pectolysis inhibition by clovamide. “Pase” = pectinase from *A. niger*. “EGCG” = epigallocatechin gallate. Pase included in all treatments, with the addition of clovamide (2 mM), EGCG (2 mM), ‘Sca6’ stage C leaf protein, or ‘Sca6’ protein in combination with either phenolic compound. Data represents two experiments (*n* = 3 from each). **(C)** Enhancement of pectinase (*A. niger*) activity by cacao stage C leaf protein (*n* = 3). No pectinase (“-Pase”) and pectinase (“+Pase”) with or without addition of ‘ICS1’ or ‘Sca6’ leaf protein. Shared letters mean no difference by Tukey-HSD at *p* < 0.0001 **(A)**, *p* < 0.05 **(B)**, or *p* < 0.0001 **(C)**. Error bars represent standard deviation.

*In vitro* proteolysis assays were performed in a reaction consisting of casein (substrate) and Proteinase K with the addition of clovamide, ‘Sca6’ stage C leaf protein, or both (Figure 5A). After protein precipitation, soluble amino acids were quantified spectroscopically by ninhydrin staining compared to a standard curve generated with glycine (*r* ^2^ = 0.9966). Clovamide alone (2 mM) inhibited proteolysis by ~27%, and a combination of ‘Sca6’ protein and clovamide inhibited proteolysis by ~40% (*p* < 0.01). ‘Sca6’ protein alone did not have an effect on proteolysis, ruling out proteolysis inhibition by components of leaf protein extract. PPO assays showed that leaf protein extracts enhanced quinone formation (Figure 4B), which is consistent with the hypothesis that the quinone product of clovamide oxidation is the cause of proteolysis inhibition.

Pectin degrading enzymes produced by plant pathogens are well known to play a major role in cell wall degradation and virulence (Prade et al., 1999; Herron et al., 2000; Herbert et al., 2003; Abbott and Boraston, 2008; Lionetti, 2015; Liu et al., 2017). In infected cacao pods, *P. megakarya* and *P. palmivora* induced expression of 27 and 40, respectively, pectinase transcripts (pectin methylesterases, polygalacturonases, and pectate lyases; Ali et al., 2017), implicating them as virulence factors in BPR development. Spence (1961) reported oxidation-dependent pectinase inhibition by cacao pod extracts. Based on this we hypothesized that clovamide is a pectinase inhibitor.

Clovamide was assayed for inhibition of pectinase from *Aspergillus niger* (Figure 5B). After incubating pectinase with pectin and various treatments, pectin remaining in solution was determined spectroscopically using ruthenium red and a standard curve generated by serial dilutions of pectin (*r* ^2^ = 0.9985). The percentage of pectin degraded was normalized to that of a pectinase only control. Epigallocatechin gallate (EGCG), a known inhibitor of pectin methylesterase, was included as a positive control (Lewis et al., 2008; Jiang et al., 2014).

Clovamide (2 mM) inhibited pectin degradation by ~55% (Figure 5B), compared to the pectinase only control (*p* < 0.0001). Combining clovamide with ‘Sca6’ protein extract resulted in a ~ 46% reduction in pectin degradation (*p* < 0.0001) but was not significantly different from the clovamide only treatment (*p* = 0.3189). EGCG (2 mM) reduced pectin degradation ~30% relative to the control (*p* < 0.0001), and the ‘Sca6’ protein plus EGCG (~26% reduction) was not significantly different from EGCG alone (*p* = 0.9129).

The addition of ‘Sca6’ protein did not enhance the effectiveness of clovamide at inhibiting pectolysis, as it did for proteolysis inhibition. ‘Sca6’ protein in the absence of clovamide, however, caused an increase in pectin degradation (~49%, *p* < 0.0001). This pectolysis-enhancing effect was measured in ‘ICS1’ and ‘Sca6’ protein extracts (Figure 5C). The cacao protein extracts alone had no detectable pectinase activity but enhanced the activity of *A. niger* pectinase (*p* < 0.0001 for ‘Sca6’ and ‘ICS1’).

Regardless of the enhanced pectolysis by cacao leaf protein, clovamide was effective at inhibiting pectin degradation substantially in the presence or absence of cacao protein extracts. Clovamide therefore probably plays an important role in defense against cell wall degradation during BPR infection.

### Clovamide Inhibits *in vitro* Growth of *Phytophthora* spp.

**Figure 6:**
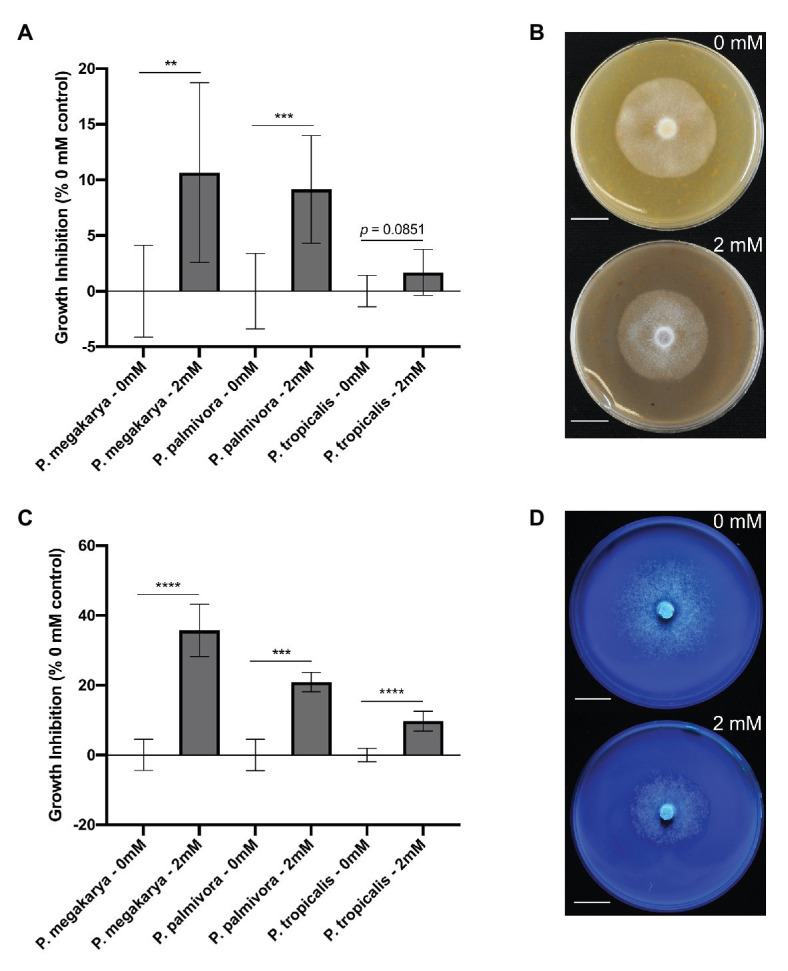
*Phytophthora* spp. growth inhibition by clovamide. **(A)** Growth inhibition (%) by clovamide of three *Phytophthora* species on V8 media. **(B)** V8 media plates with *P. megakarya* mycelia with 0 mM clovamide (top) and 2 mM clovamide (bottom). **(C)** Growth inhibition (%) by clovamide of three *Phytophthora* species on semi-synthetic Henniger/Casein/Pectin (“HenCasPec”) media. **(D)** HenCasPec media plates with *P. megakarya* mycelia with 0 mM clovamide (top) and 2 mM clovamide (bottom), stained with calcofluor and photographed under 365 nm excitation for contrast between white mycelia and white media. Growth inhibition data from two experiments (*n* = 4 from each), except *P. palmivora* on HenCasPec, which represents one experiment. *p* < 0.01 (^**^), *p* < 0.001 (^***^), *p* < 0.0001 (^****^), *t*-test. Error bars in **(A)** and **(C)** represent standard deviation.

The ability of clovamide to inhibit growth of three major BPR-causing pathogens (*Phytophthora* spp.) was assayed. Growth inhibition was first performed on V8-agar media (Jeffers and Martin, 1986) (Figures 6A,B). Clovamide (2 mM) inhibited growth of *P. megakarya* (10.65 ± 3.203%, *p* < 0.01) and *P. palmivora* (9.149 ± 2.088%, *p* < 0.001) but did not display significant inhibition of *P. tropicalis* (1.687 ± 0.8850%, *p* = 0.0851).

Since clovamide inhibited proteolysis and pectolysis *in vitro* (Figures 5A,B), we hypothesized that nitrogen source and pectin matrix may have an effect on clovamide’s growth inhibition potential. Thus, a synthetic media was prepared based on Henniger Synthetic Media (Henniger, 1963), but with all nitrogen sources removed and replaced by casein protein (8 g/L). Pectin was included (0.1% w/v) as well to produce Henniger/Casein/Pectin media. Under these conditions, there was indeed a significant effect and clovamide inhibited growth of all pathogens as follows (Figures 6C,D): *P. megakarya* (35.71 ± 3.098%, *p* < 0.0001), *P. palmivora* (20.89 ± 2.644%, *p* < 0.001), and *P. tropicalis* (9.725 ± 1.200%, *p* < 0.0001).

### Metabolomics of Fruit Peel Extracts Reveals Accumulation of Sulfated HCAAs Exclusively in ‘Sca6’

**Figure 7:**
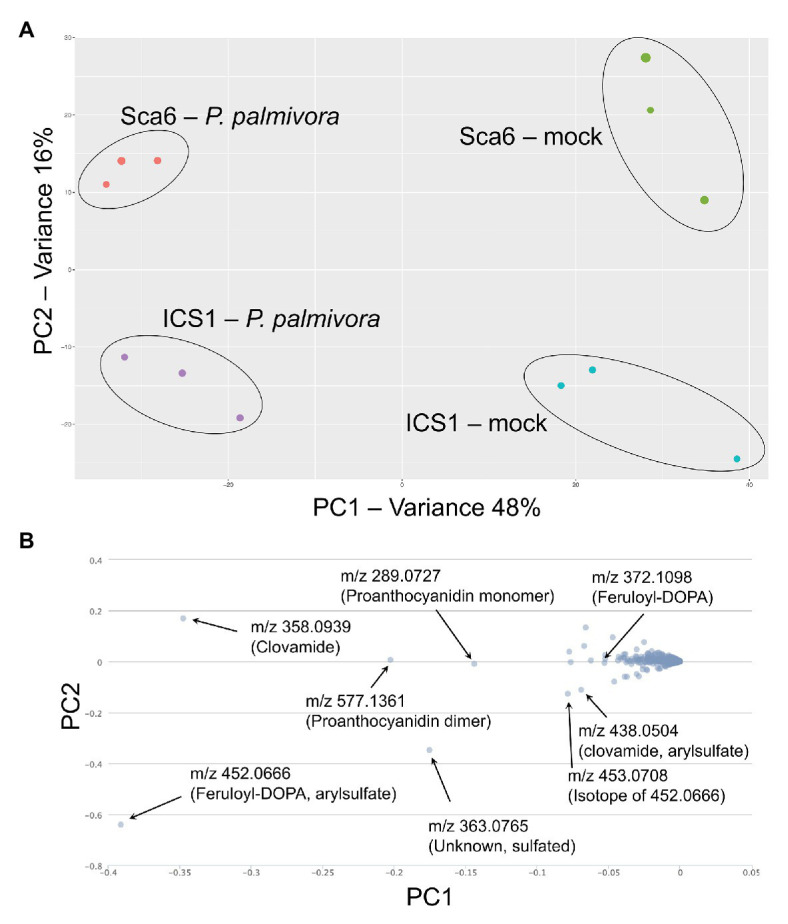
LC-MS metabolomics of pod (fruit) infection by *P. palmivora* overview. **(A)** Principle Components Analysis (PCA) of LC-MS data, *P. palmivora*-infected or mock inoculated. **(B)** Loading chart showing contribution of individual metabolite features to each principle component. Greater deviation from zero on either axis represents a larger contribution to the respective principle component. Major contributors to each principle component are indicated with median mass to charge ratios (m/z) and putative annotations. All m/z shown are suspected molecular ions ([M-H]^−^) except one indicated as an isotope.

Fruit peels (exocarp and ~2 mm mesocarp) from both ‘Sca6’ and ‘ICS1’ contained clovamide, which suggests that clovamide is not a distinguishing factor in fruit (pod) resistance between the two genotypes (Figure 3B). Therefore, to determine if other metabolites differentiate the two genotypes in this tissue, untargeted LC-MS/MS was performed on fruit peel extracts. Fruits (4–5 month old) were wounded and inoculated with mycelium plugs of *P. palmivora* “GhER1349” or sterile media plugs (mock inoculated control). Metabolite extraction and LC-MS/MS were performed 72 h after inoculation. Sample groups separated distinctly in principle component analysis (PCA) of the LC-MS data, with PC1 separating by treatment (mock vs. *P. palmivora*) and PC2 separating by genotype (‘Sca6’ vs. ‘ICS1’; Figure 7A). The associated loading chart (Figure 7B) shows contributions of individual metabolites to each principle component in the PCA plot.

Data filtering was performed on mock-inoculated ‘Sca6’ and ‘ICS1’ samples as previously described for leaf to detect differences in basal (not infection-induced) metabolites. Of metabolite features >5-fold higher (*p* < 0.05) in ‘Sca6’ (mock) vs. ‘ICS1’ (mock), the two most abundant ‘Sca6’ metabolites (m/z 452.07 and 438.05, [M-H]^−^) had striking fold-changes (7,070‐ and 7,152-fold higher in ‘Sca6’ mock vs. ‘ICS1’ mock). The same compounds are indicated in the loading chart (Figure 7B). The compounds were manually annotated as arylsulfated feruloyl-DOPA and arylsulfated clovamide, respectively, based on shared MS/MS fragments with the base compounds feruloyl-DOPA and clovamide, and the presence of a fragment of m/z 79.96, which indicates a sulfate group on an aromatic ring (Weidolf et al., 1988). MS/MS spectra for the two sulfated HCAAs and other metabolites indicated in Figure 7B can be found in Supplementary Data File 3.

Aryl-sulfated feruloyl-DOPA (m/z 452.07, [M-H]^−^) was also in the top 30 metabolite features identified in the leaf metabolomics experiment (Table 1, metabolite feature S-25), but was considerably less abundant than clovamide. In fruit peel, aryl-sulfated feruloyl-DOPA (m/z 452.07, [M-H]^−^) was overall the most abundant signal in ‘Sca6’ (mock treatment) and aryl-sulfated clovamide (m/z 438.05, [M-H]^−^) was the ninth most abundant.

**Figure 8:**
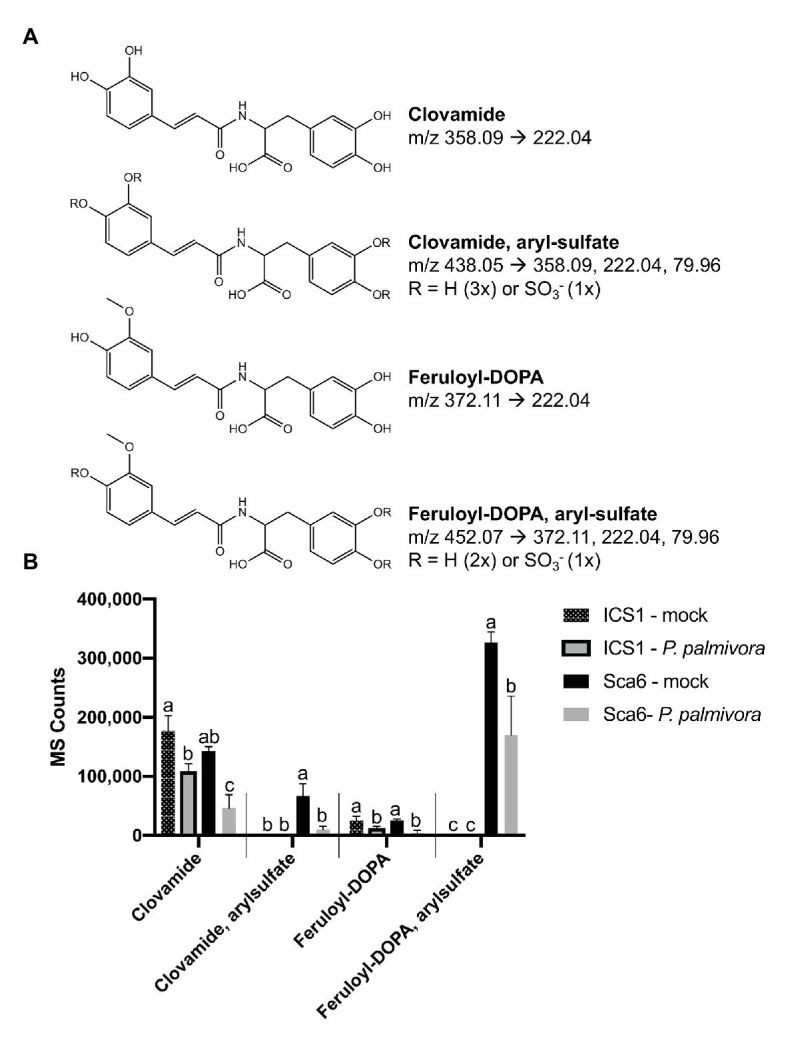
Changes in four HCAAs during fruit/pod infection by *P. palmivora*. **(A)** Putative structures of two sulfated HCAAs, feruloyl-DOPA, and clovamide. Parent ion m/z ([M-H]^−^) and diagnostic MS/MS fragments shown. **(B)** Metabolite abundance of the same four HCAAs in ‘ICS1’ and ‘Sca6’ fruit/pod tissue, mock inoculated or infected by *P. palmivora*. MS Counts represent mass spectrometer signal intensity of peaks integrated in XCMS Online (Tautenhahn et al., 2012). Shared letters mean no difference by Tukey-HSD (*p* < 0.05, *n* = 3). Error bars represent standard deviation.

Clovamide, aryl-sulfated clovamide, feruloyl-DOPA, and aryl-sulfated feruloyl-DOPA (Figure 8A) were not induced by infection, but rather a significant reduction was observed for all four HCAAs upon infection by *P. palmivora* (Figure 8B). A proanthocyanidin monomer (catechin/epicatechin; m/z = 289.07, [M-H]^−^) and a proanthocyanidin dimer (m/z 577.14, [M-H]^−^), also indicated in Figure 7B, decreased with infection but were not different between genotypes (Supplementary Figure 3).

A follow-up LC-MS/MS experiment was performed to test if the sulfated HCAAs were induced by wounding in the mock inoculated control treatment. Extracts of three unwounded fruits were compared to three wounded fruits of both genotypes. Aryl-sulfated feruloyl-DOPA (m/z 452.07, [M-H]^−^) was again the most abundant signal in ‘Sca6,’ and neither of the sulfated HCAAs were induced by the wounding treatment (Supplementary Figure 4). In contrast to leaves, grinding of fruit peel in water did not result in more supernatant browning in ‘Sca6’ than ‘ICS1’ (Supplementary Figure 5).

Taken together, two putative sulfated HCAAs, aryl-sulfated feruloyl-DOPA (m/z 452.07, [M-H]^−^) and aryl-sulfated clovamide (m/z 438.05, [M-H]^−^), constitute the major differences between ‘Sca6’ and ‘ICS1’ in fruit peel. The two compounds decrease during infection similarly to clovamide.

## Discussion

Our findings that clovamide and sulfated HCAAs accumulate to very high levels in a pathogen tolerant genotype (‘Sca6’) as compared to a pathogen susceptible genotype (‘ICS1’) implicated clovamide as a potentially important contributor to plant defense in cacao. We characterized several properties of clovamide consistent with this hypothesis and found that clovamide contributes to PPO-mediated enzymatic browning, inhibits pectolysis and proteolysis, and inhibits the growth of three *Phytophthora* species that infect cacao. Taken together, our data demonstrate that clovamide is an important metabolite in the plant’s arsenal of defense compounds, along with other HCAAs.

Our approach spanning untargeted metabolomics to functional analysis of clovamide’s role in defense provides a straightforward roadmap for future experiments. Metabolite identification from LC-MS/MS data is notoriously difficult, especially so in plants, which produce a remarkable diversity of secondary or specialized metabolites that are not yet searchable in spectral databases (Shahaf et al., 2016; Perez de Souza et al., 2017; Viant et al., 2017). To address this bottleneck, we applied simple filters to our LC-MS/MS data to focus annotation efforts on high priority metabolite features: >5-fold higher in ‘Sca6,’ *p* < 0.05, 30 most abundant in ‘Sca6’. Since most of these features were annotated as HCAAs, we expanded our scope and performed a semi-targeted search for HCAAs using calculated masses and predicted fragment masses. Of the HCAAs higher in ‘Sca6,’ we selected the most abundant, clovamide, for synthesis, targeted quantification, and functional characterization.

### Clovamide, a Novel Source of *Phytophthora* spp. Resistance and a Potential Breeding Target for Cacao

The cacao genotype ‘Sca6’ has been an important source of resistance to BPR for breeders, and has served as an international reference for resistance (Tahi et al., 2000; Lachenaud et al., 2001; Pokou et al., 2008; Akaza et al., 2009; Thevenin et al., 2012). The molecular and biochemical underpinnings of its resistance, however, have remained elusive. This work demonstrates that clovamide content of ‘Sca6’ is likely a major factor in the observed difference in leaf resistance compared to ‘ICS1.’

‘Sca6’ has also been heavily relied upon as a source of resistance to Witches’ Broom Disease (WBD, caused by *Moniliophthora perniciosa*; Pires et al., 2012; Feitosa Jucá Santos et al., 2014). Although the effect of clovamide against *M. perniciosa* was not evaluated, the potential for this compound’s importance in WBD resistance exists. Clovamide has previously been reported as an inhibitor of influenza A subtype H5N1 (virus) and *T. evansi* (protozoa; El-Sharawy et al., 2017), and *H. pylori* (Niehues et al., 2011), and may therefore have broad spectrum antimicrobial activity.

Progress in breeding of cacao has been hampered by long generation times, self-incompatibility, complex inheritance of oligogenic resistance, limited resources of breeding programs, and reliance on a relatively small proportion of available genetic diversity (Lanaud et al., 2009, 2017; Bekele and Phillips-Mora, 2019). Significant strides have been made in genetic mapping of resistance QTL associated with BPR and WBD resistance (Lanaud et al., 2009; Akaza et al., 2016; Barreto et al., 2018). Application of microsatellite markers and genomic selection has shown promise for accelerating breeding of cacao (Schnell et al., 2007; McElroy et al., 2018). However, genetic markers, while useful, are not yet understood at a mechanistic level. Incorporation of metabolite markers such as leaf clovamide as early selection criteria in breeding programs would be beneficial, especially since such anti-microbial agents can be directly detected as opposed to genetic markers that are merely linked to resistance. Furthermore, high/low abundance of clovamide in methanolic extracts of leaf tissue can easily be qualitatively scored using TLC (Figure 3C). This simple, rapid, and inexpensive TLC marker should be tested in cacao populations with ‘Sca6’ parentage (and others) to validate its potential utility as a breeding marker for BPR and WBD resistance. High performance – liquid chromatography coupled with a diode array detector (HPLC-DAD) is quantitative and more sensitive than TLC and may also be employed where available. The compound is constitutively produced in ‘Sca6’ leaves, which means that no induction by pathogen inoculation is required to perform this screen, further simplifying the implementation of this marker.

### Polyphenol Oxidase and Cacao Resistance

Polyphenol oxidase (PPO) activity has been reported in cacao leaf, pod (fruit), and seed tissues towards the substrates catechol, L-DOPA, dopamine, 4-methylcatechol, epicatechin, and catechin (Lee et al., 1991; Okey et al., 1997; Simo et al., 2011; Macedo et al., 2016; Ondobo et al., 2017; Fantinato et al., 2018). PPO activity has also been reported to increase upon infection by *P. palmivora* in cacao stems (Okey et al., 1997) and *P. megakarya* infection in pods (Simo et al., 2011). Spence (1961) proposed that the higher *P. palmivora* resistance of ‘Sca6’ pods compared to those of ‘ICS1’ was due to faster enzymatic browning, although specific metabolites involved in browning were not determined. In the same study, cacao pods infected with *P. palmivora* showed increases in lesion size and a reduction in lesion browning in a low oxygen atmosphere. While the evidence suggests that PPO-mediated browning is an important factor in BPR resistance, characterization of individual *o*-diphenol substrates found at appreciable concentrations in cacao tissue is limited to catechin and epicatechin (Simo et al., 2011; Macedo et al., 2016). To our knowledge, clovamide has not been characterized as a substrate for cacao PPO prior to this study. We were able to demonstrate that clovamide and related HCAAs decreased during *P. palmivora* infection (Figure 8B) as did a proanthocyanidin monomer and dimer (Supplementary Figure 3), a pattern consistent with PPO-mediated oxidation.

Special care should be taken when evaluating the contribution of PPO substrates to resistance. Growth inhibition of a PPO substrate such as clovamide *in vitro* may be difficult to relate to growth inhibition of a pathogen *in planta*. A PPO substrate could lack direct toxicity to a pathogen but slow nutrient acquisition by inhibition of excreted digestive enzymes, or act as a physical barrier through oxidative coupling with cell wall components. The higher level of growth inhibition in synthetic Henniger/Casein/Pectin media compared to V8-agar (Figures 6A,C) may be due to such effects on nutrient acquisition. Henniger/Casein/Pectin media lacks amino acids, nitrate, and ammonium, with casein protein as the sole source of nitrogen. Furthermore, the protein is in a matrix of pectin. In order to acquire nitrogen for growth, the pathogens must rely on their ability to access protein in a pectin matrix and degrade the protein with excreted proteases. Protease and pectinase inhibition by clovamide (Figures 5A,B) provides an explanation for the higher degree of growth inhibition measured in Henniger/Casein/Pectin media compared to V8-agar. If a PPO substrate requires PPO activity to undergo oxidation to quinone, and if it lacks direct toxicity to a pathogen, *in vitro* growth inhibition assays may underestimate its contribution to resistance *in planta*. Clovamide can undergo non-enzymatic oxidation on a short time scale (Figure 4B; 10-min assay), and browning of media containing clovamide was visible by the end of growth inhibition assays (Figure 6B). This property of clovamide may be what allows for the observed growth inhibition *in vitro*. For other PPO substrates, it may be necessary to incorporate PPO activity into the assay to facilitate quinone formation.

Clovamide exhibited the highest degree of growth inhibition in *P. megakarya*, followed by *P. palmivora*, and *P. tropicalis* (Figures 6A,C). This pattern is inversely related to the growth rates of the three pathogens *in vitro* (Supplementary Figure 6). *P. tropicalis* grows the fastest *in vitro* and *P. megakarya* grows the slowest. The relatively lower degree of growth inhibition of *P. tropicalis* by clovamide may be due to its growth outpacing the oxidation of clovamide in the assay. This may be another factor to consider when evaluating growth inhibition by PPO substrates.

### Tissue Specificity of HCAA Accumulation

This work highlights the importance of considering tissue-specific resistance mechanisms. While ‘Sca6’ accumulates far more clovamide than ‘ICS1’ in leaf tissue, both genotypes produce substantial amounts of clovamide in fruit peel. In fruit peel, ‘Sca6’ distinguishes itself from ‘ICS1’ by accumulating sulfated HCAAs. Lechtenberg et al. (2012) reported clovamide accumulation in pod husk but not in leaf, similar to what we observed in ‘ICS1’ (Figure 3B), although the authors did not specify genotype or leaf developmental stage.

Leaf resistance has been demonstrated to correlate reasonably well with field resistance measured as infected pod count (Tahi et al., 2000, 2006). This could be due to shared resistance mechanisms across tissues, reduced secondary inoculum produced during leaf infection for subsequent pod infection, or both. Leaf and pod tissues could also have distinct but related mechanisms of resistance, such as HCAA accumulation in leaves and sulfated HCAA accumulation in pods, meaning that QTL studies of disease resistance may reveal different loci depending on which tissue’s resistance is used as the phenotype. For example, QTL analysis of leaf resistance may reveal biosynthetic genes in the pathway up to clovamide (e.g., BAHD-acyltransferases; Sullivan and Bonawitz, 2018), whereas analysis of pod phenotypes may reveal genes involved in sulfation of HCAAs (e.g., sulfotransferases; Hirschmann et al., 2014). Supplementary Figure 7 presents hypothetical biosynthetic pathways to clovamide and sulfated clovamide, which may guide candidate gene selection in future QTL analyses. Considering tissue-specific resistance traits further complicates the already challenging task of breeding cacao but may be advantageous in addition to selection for markers in QTL with consensus across tissues.

The role of sulfated HCAAs in ‘Sca6’ pod remains unclear. The compounds may be undergoing non-enzymatic or PPO-mediated oxidation during BPR infection by *Phytophthora* spp., which is supported by their reduction during infection by *P. palmivora* (Figure 8B). Regardless of the position of the sulfate group in the aryl-sulfated clovamide (Figure 8A), the molecule still has at least one unsulfated *o*-diphenol moiety, making it a candidate PPO substrate. The aryl-sulfated feruloyl-DOPA, depending on position of the sulfate (Figure 8A), may or may not have one exposed *o*-diphenol group to serve as a substrate for PPO. Sulfate esters of aromatic rings are known to be labile, however, and may undergo hydrolysis non-enzymatically or by the action of arylsulfatases, which may liberate *o*-diphenol moieties from the sulfated forms (Ragan, 1978; Simpson and Widlanski, 2006; Raghuraman et al., 2007; Mattarei et al., 2015). In contrast to this hypothesis and observations by Spence (1961), enhanced browning was not observed in ground ‘Sca6’ pod tissue compared to ‘ICS1’ (Supplementary Figure 5). Perhaps the sulfate group somehow favors covalent cross linking with chemical species other than proteins, which is associated with melanization and browning. For example, HCAA binding at the cell wall by covalent binding to lignin would not necessarily manifest as browning. HCAA deposition at cell walls has been observed in wheat during infection by *Fusarium graminearum*, which resulted in cell wall thickening associated with resistance (Gunnaiah et al., 2012).

The role these sulfated HCAAs is not clear at this point. However, their structural similarity to clovamide, high abundance in the tolerant ‘Sca6,’ and virtual absence in the susceptible ‘ICS1’ suggest that they may be important factors in defense against *Phytophthora* spp. This work demonstrated that clovamide is a growth inhibitor of *Phytopthora* spp. as well as a proteolysis and pectolysis inhibitor. Similar assays with purified forms of these sulfated HCAAs or genetically modified plants with modulated accumulation of sulfated HCAAs will be required to unambiguously test their role in conferring resistance to *Phytophthora* spp.

### Antimicrobial Activity/Enzyme Inhibition of Clovamide and Potential Mechanisms Thereof

As mentioned previously, clovamide has been reported as a potent inhibitor of influenza A subtype H5N1 (virus) and *T. evansi* (protozoa; El-Sharawy et al., 2017). Niehues et al. (2011) determined that clovamide can partially inhibit adherence of *Heliobacter pylori* to gastric epithelial cells, an important factor in *H. pylori* virulence. In this work, we report growth inhibition of three species of *Phytophthora*. This broad-spectrum antimicrobial activity may suggest a generic mechanism, such as *o*-diphenol oxidation to quinone and indiscriminate covalent cross-linking to reducing groups (-SH, -NH_2_) in proteins, rather than a specific one such as selective binding to the active site of an essential enzyme. If this were true, similar broad-spectrum enzymatic inhibition might also be expected. Clovamide inhibits alfalfa proteases (Sullivan and Zeller, 2013), proteinase K from *Tritirachium album* (Figure 5A), and pectinase from *Aspergillus niger* (Figure 5B). While it is unclear as of yet if clovamide is acting directly on these enzymes to inhibit them, or if it is cross-linking protein and pectin substrates making them less digestible, the current evidence suggests a generic mechanism.

Cacao leaf protein extracts enhanced the activity of pectinase from *A. niger* but had no detectable pectinase activity on their own (Figure 5C). The pectinase activity enhancement could be due to pectin methylesterase or expansin activity in the cacao protein extracts. De-methylesterification should enhance binding to ruthenium red used in the assay and subsequent pectin precipitation (Lionetti, 2015), which would manifest as an apparent increase in pectin concentration relative to pectin solution without cacao protein. This was not observed in our experiment so pectin methylesterase activity is not likely responsible for the observed effect. Expansin activity cannot be ruled out, however. Cucumber alpha-expansin protein has been reported to enhance fungal pectin lyase activities (Wei et al., 2010), and the cacao leaf protein used in the assay was from actively expanding “stage C” leaves. Endogenous plant proteins such as pectin methylesterases and expansins, while important for normal development, may inadvertently assist pathogen-excreted pectin degrading enzymes. The same could be said for endogenous plant proteases. Deployment of a broad-spectrum enzyme inhibitor such as clovamide may be an effective way to inhibit enzymatic activities, plant‐ or pathogen-derived, that facilitate pathogen progression through plant tissue.

### Potential for Genetic Engineering of Clovamide Biosynthesis in Other Crops and Limitations

The genus *Phytophthora* contains over 100 species, many of which are plant pathogens with broad host ranges (Thines, 2014). Introducing clovamide biosynthesis into other crops affected by *Phytophthora* spp. may be an effective approach to enhance resistance. Furthermore, increased clovamide content in food may provide human health benefits due to its anti-inflammatory and neuroprotective properties (Park et al., 2007, 2017; Fallarini et al., 2009; Zeng et al., 2011; Kolodziejczyk-Czepas et al., 2017; Tsunoda et al., 2018). The recent cloning and characterization of a hydroxycinnamoyl-CoA:L-DOPA hydroxycinnamoyl transferases (HDT) from red clover (*Trifolium pratense*) capable of catalyzing the formation of clovamide from caffeoyl-CoA and *L*-DOPA (Sullivan and Bonawitz, 2018; Bouchez et al., 2019) means it is now conceivable to transfer clovamide biosynthesis into other crops *via* genetic engineering.

There are potential limitations to use of clovamide as a means of enhancing disease resistance or health-promoting properties of crops, however. Clovamide has been previously studied in cacao and cocoa products (chocolate and cocoa powder) with respect to its antioxidant activity (Sanbongi et al., 1998) but also for its contribution to astringency (Stark and Hofmann, 2005). Negative effects on flavor attributes of other crops may make high-clovamide genotypes less desirable to consumers. Furthermore, clovamide will likely enhance browning associated with *o*-diphenol oxidation. Browning in fruits and vegetables is generally viewed as undesirable, leading to rejection by consumers and considerable post-harvest losses (Queiroz et al., 2008). This strong preference for non-browning produce has even led to the development and approval PPO-silenced apple and potato varieties with reduced browning (Waltz, 2015a,b).

It is unclear at this point if breeding for high leaf clovamide content in cacao will be accompanied with increases in seed content associated with undesirable flavor. Arlorio et al. (2008) reported reductions (up to ~59%) in clovamide content of cacao seeds during roasting, an important step in the post-harvest processing of cacao seeds into edible cocoa. Perhaps special attention to post-harvest processing conditions can ameliorate negative flavor impacts of clovamide in cacao.

## Materials and Methods

### Untargeted Metabolomics of Leaf Tissue: Liquid Chromatography – Mass Spectrometry

Stage C (mid-stage of development) leaf tissue (Mejía et al., 2012) was collected from ‘Sca6’ and ‘ICS1’ trees grown in a greenhouse as previously described (Swanson et al., 2008). Three to five leaves from different branches on a tree were combined per sample and flash frozen in liquid nitrogen after midrib removal. Three clonally propagated trees were sampled per genotype. Tissue was ground in liquid nitrogen and extracted with 80% methanol and 0.1% formic acid in water (v/v), using a 3:1 solvent to tissue ratio (μl:mg) as previously described (De Vos et al., 2007). Genistein was included as an internal standard (2.5 μg/ml; Calderón et al., 2009). Extracts were filtered using 0.2 μm spin columns (Norgen Biotek Corp. Cat. #40000) before LC-MS/MS analysis. LC-MS grade solvents were used.

Liquid Chromatography–Mass Spectrometry (LC-MS/MS) was performed at the Pennsylvania State University Metabolomics Facility at the Huck Institutes of the Life Sciences. Reverse phase HPLC was performed to separate samples (5 μl) with a Prominence 20 UFLCXR system (Shimadzu, Columbia, MD) equipped with a Waters (Milford, MA) BEH C18 column (100 × 2.1 mm, 1.7 μm) maintained at 55°C, using a 20-min aqueous acetonitrile gradient with a flow rate of 250 μl/min. HPLC grade water with 0.1% formic acid was Solvent A and HPLC grade acetonitrile with 0.1% formic acid was solvent B. The HPLC gradient was as follows: 3% B increased to 45% B from 0 to 10 min, 45% B to 75% B from 10 to 12 min, hold at 75% B from 12 to 17.5 min, return to initial conditions (3% B). A Duospray™ ion source was used to deliver eluate to a 5600 (QTOF) TripleTOF Mass Spectrometer (both AB Sciex, Framingham, MA). The ion source was operated in ESI mode with a capillary voltage of 4.5 kV (negative ion mode), with a declustering potential of 80 V. The mass spectrometer was operated in Information Dependent Acquisition (IDA) mode with a 100 ms survey scan from 50 to 1,500 m/z. Up to 20 product ion scans (MS^2^) were performed per duty cycle using a collision energy of 50 V with a 20 V spread.

Liquid chromatography–mass spectrometry (LC-MS) data were analyzed using XCMS Online (peak alignment and Welch’s *t*-test for each metabolite feature; Tautenhahn et al., 2012). MS-Dial (v2.58) was used to extracts MS/MS (MS^2^) spectra for metabolites of interest (Tsugawa et al., 2015).

Metabolite features were putatively annotated by searching in the METLIN database (Guijas et al., 2018) or based on matching MS/MS fragments to those previously reported in the literature (Arlorio et al., 2008; Alonso-Salces et al., 2009; Szajwaj et al., 2011; Oracz et al., 2019). Alkyl‐ and aryl-sulfated derivatives were annotated based on ~96.96 Da and ~79.96 Da MSMS fragments, respectively, as described by Weidolf et al. (1988).

### Semi-Targeted Search for HCAAs in LC-MS/MS Dataset

A semi-targeted search for HCAAs was performed by generating a library of hypothetical HCAA MS^1^ parent ion masses and MS/MS fragment masses for 250 compounds. In order for a compound in our LC-MS/MS data to be considered a match, it must match the parent ion ([M-H]^−^) in MS^1^ (±0.05 Da) and contain at least one of the diagnostic MS/MS fragments. This library consisted of compounds with hydroxycinnamoyl moiety in an amide bond with an amine moiety. Hydroxycinnamoyl moieties (cinnamoyl, coumaroyl, caffeoyl, feruloyl, and sinapoyl) in combination with all proteogenic amino acids, GABA, DOPA, the decarboxylated forms of the amino acids, and several polyamines (spermine, spermidine, cadaverine, and putrescine) gave a list of 250 predicted compounds. MS/MS fragments were calculated based on characteristic fragmentation patterns observed near the amide bond in caffeoyl-DOPA (clovamide), as described by Arlorio et al. (2008), (Figure 2A).

Hydroxycinnamic acid amides (HCAAs) containing a decarboxylated amino acid (agmatine, tyramine, etc.) as the amine moiety were considered artifacts of in-source fragmentation if they co-eluted with the corresponding carboxylated form. For example, coumaroyl-tyramine was initially identified but later ruled out as an ionization artifact due to in-source decarboxylation of coumaroyl-tyrosine, which had the same retention time.

The isobaric monodeoxyclovamides coumaroyl-DOPA and caffeoyl-tyrosine could not be differentiated by MS/MS fragments, since two features in the LC–MS/MS dataset had all four diagnostic MS/MS fragments for both compounds (see Supplementary Data File 2). The two compounds were therefore annotated based on relative retention times for the two compounds reported by (Bouchez et al., 2019).

### Synthesis of Clovamide

*Trans*-Clovamide was synthesized using methods described by Xie et al. (2013), starting with *trans*-caffeic acid (Cayman Chemical 70602) and *L*-DOPA methyl ester (Sigma-Aldrich D1507). The product was dissolved in DMSO-d_6_ and analyzed (^1^H NMR) on a Bruker AVIII-HD-500. Retention time and absorbance spectrum of the product were compared with commercial clovamide (Cayman Chemical 16138) using HPLC-DAD.

### HPLC-DAD for Targeted Analysis of Clovamide

Stage A/B, C, and D/E leaves (Mejía et al., 2012) were collected from clonally propagated ‘Sca6’ and ‘ICS1’ trees grown as previously described (Swanson et al., 2008). Five trees of each genotype were sampled. Three to five pooled leaves of the same developmental stage from the same tree constituted a replicate. Cacao pods (4–5 month old fruits) were kindly provided by next day shipping by Dr. Ricardo Goenaga (USDA-ARS, Mayaguez, PR). Three pod peel samples from each genotype were collected using a potato peeler (exocarp plus ~2 mm mesocarp). Frozen, ground tissue was extracted as above except a 20 μl:1 mg solvent:tissue ratio was used and genistein was not included. 100 μl of extract was dried in a SpeedVac (Savant) and re-dissolved with sonication in 300 μl 10:90:0.1 methanol:water:formic acid (v/v/v) with 2.5 μg/ml rosmarinic acid as an internal standard (Cayman Chemical 70900). Extracts were filtered using 0.2 μm spin columns (Norgen Biotek Corp. Cat. #40000) before HPLC-DAD analysis.

High-performance liquid chromatography (HPLC) was performed on an Agilent 1260 Infinity system with an Agilent Poroshell EC-C18 column (150 × 3 mm, 2.7 μm) maintained at 40°C, using a 45-min aqueous methanol gradient with a flow rate of 0.5 ml/min. HPLC grade water with 0.1% formic acid was solvent A and HPLC grade methanol with 0.1% formic acid was solvent B. The solvent gradient was as follows: 5% B to 95% B from 0 to 30 min, hold at 95% B from 30 to 40 min, return to initial conditions (5% B) for 5 min. Sample injection volume was 10 μl. Absorbance at 320 nm was used to detect clovamide. Comparison of retention time and absorbance spectra to that of pure clovamide was used for compound identification. Solutions of clovamide (0.05, 0.01, 0.002, 0.0004, and 0.00008 mg/ml) were analyzed to generate a standard curve for quantification in cacao extracts.

### Thin-Layer Chromatography for Clovamide Detection

Three stage C leaf samples from ‘Sca6’ or ‘ICS1’ were extracted as described above (3,1 solvent,tissue ratio). Ten microliters of the leaf extract or clovamide standard (10 μM) were loaded per lane (1 cm width) on 10 cm × 10 cm HPTLC Silica gel 60 plates (Merck). Plates were developed with 100:10:10:10 ethyl acetate:glacial acetic acid:formic acid:water (v:v:v:v) until the solvent front reached ~90% of the plate length. Plates were photographed under 365 nm UV excitation.

### Protein Extraction From Cacao Leaf

The protein was extracted from stage C leaves of three ‘Sca6’ and three ‘ICS1’ trees according to Pirovani et al. (2008), with the addition of 2 μl/ml Plant Protease Inhibitor Cocktail (Sigma P9599) in the extraction buffer. Metabolites were removed by buffer exchange in 10 kDa cutoff centrifugal filters (Amicon® Ultra – Merck Millipore Ltd.) using protein storage buffer (50/50 v/v glycerol/Tris-HCl, 20 mM, pH 7.5). The protein was quantified using the Qubit™ Protein Assay Kit (ThermoFisher Q33211), diluted to 150 ng/μl in protein storage buffer, and stored at −20°C for PPO and pectolysis assays. Protein was precipitated in 90% ice-cold ethanol and re-suspended in 2-(*N*-morpholino) ethanesulfonic acid (MES) buffer (0.2 M, pH 6.5) for use in proteolysis assays. This extra step was included to ensure complete removal of protease inhibitors from the protein extraction.

### Polyphenol Oxidase Activity Assay

Polyphenol oxidase (PPO) activity assays were performed as described by Sullivan and Foster (2013) using the 2-nitro-5-thiobenzoic acid (TNB) quinone trap assay (Esterbauer et al., 1977). Each reaction master mix contained 965 μl McIlvaine’s buffer (pH 7), 20 μl TNB solution, 10 μl clovamide (50 mM in DMSO), 5 μl cacao leaf protein (‘Sca6’ or ‘ICS1’, 150 ng/μl). From this master mix, 200 μl were dispensed in a microtiter plate (3 technical replicates per reaction). Reactions were incubated 10 min at 25°C and Abs_412 nm_ was measured every 30 s. The slope of the linear regression was calculated in Microsoft Excel and converted to nmol quinone/min using the conversion factor 91.0 nmol/Abs_412 nm_ (Esterbauer et al., 1977) adjusted for to the pathlength in the microtiter well (0.6 cm). PPO activity was measured for three biological replicates (separate clonally propagated trees) of ‘Sca6’ and ‘ICS1.’ Controls were performed without clovamide (DMSO only) to account for potential metabolite carryover from protein extractions and without protein (buffer only) to account for non-enzymatic oxidation of clovamide. Catalase (Thermo Scientific J12885-03) was included (280 U/ml) in one iteration of the experiment to account for peroxidase activity (Gertzen and Escobar, 2014). Kojic acid (Cayman Chemical 22712), a PPO inhibitor, was included in some assays at 1 mM or 5 mM (Gertzen and Escobar, 2014). Reactions including SDS (0.25% w/v) were also performed to test for latent PPO activity (Moore and Flurkey, 1990). ANOVA was performed and means were separated by pairwise *t*-tests (two-sided).

### Browning Assay

A cork borer (9 mm) was used to cut out leaf disks from stage C leaves, which were placed in 2 ml screw cap tubes with 1 ml water and stainless-steel beads (1 × 3 mm and 2 × 1 mm). Leaf disks were ground in a TissueLyser (Qiagen) for 8 min at 30 Hz and incubated at 25°C (total 30 min since start of grinding). Samples were centrifuged (14,000 × *g*, 5 min) and absorbance (418 nm) of the supernatant was measured in a spectrophotometer. Four leaves from different trees were analyzed from each genotype. Clovamide (0.077% v/v of 100 mM stock in DMSO, or ~27.7 μg per sample) or DMSO (0.077%) were added to samples before grinding to determine the effect of clovamide on browning. This amount was calculated based clovamide quantification data (HPLC-DAD) and the average mass of a leaf disk to determine the average amount of clovamide per ‘Sca6’ leaf disk.

The wavelength for analysis was determined by performing the assay in the presence or absence of kojic acid (5 mM), a PPO inhibitor. Absorbance intensities (325–600 nm scan) of the kojic acid treated samples were subtracted from those of samples ground in water to identify ~418 nm as the absorbance maximum for PPO-mediated browning (mean Abs_max_ from three ‘Sca6’ and three ‘ICS1,’ see Supplementary Figure 2).

### Note on Clovamide Concentration Used in Proteolysis, Pectolysis, and Growth Inhibition Assays

In order to assess clovamide’s ability to inhibit digestive enzyme function and *Phytophthora* spp. growth *in vitro*, it was important to test clovamide at a concentration that is physiologically relevant. Average leaf water content of stage A/B, C, and D/E leaves (79.8, 83.0, and 63.7% w/w) was determined and combined with clovamide content determined by HPLC (mg/g tissue) to estimate molarity in the tissue. Leaf clovamide content ranged from 0.14 to 0.28 mM in ‘ICS1’ and 3.61 to 11.24 mM in ‘Sca6’ A clovamide concentration of 2 mM was chosen because it is in the physiologically relevant range and could be delivered as a 100 mM stock solution in DMSO while maintaining a low DMSO concentration (2% v/v) in the final media or buffer. Giannakopoulou et al. (2014) reported no toxicity of DMSO to other *Phytophthora* species at concentrations less than 2.5% (v/v).

### Proteolysis Assay

Proteolysis assays were adapted from Sullivan and Foster (2013) and used casein (Sigma C7078) digestion by Proteinase K from *T. album* (Sigma P8044) as a model reaction. Each 100 μl reaction consisted of 84 μl MES buffer (0.2 M, pH 6.5), 12 μl casein (0.833 mg/ml stock in MES for 0.1 mg/ml final concentration), 1 μl ‘Sca6’ cacao leaf protein (40 ng/μl in MES), 2 μl clovamide (100 mM stock in DMSO), and 1 μl Proteinase K (1 mg/ml stock in MES). Negative controls were run with buffer in place of enzymes (‘Sca6’ protein or Proteinase K) and DMSO in place of clovamide. Reactions were incubated 18 h at 37°C. After incubation, un-digested protein was precipitated by adding 900 μl cold ethanol (−20°C) followed by centrifugation for 10 min at 14,000 × *g* (4°C). To quantify soluble amino acids in the supernatant, 750 μl was mixed with 150 μl ninhydrin solution (3.5 mg/ml in ethanol), heated for 10 min at 90°C in a screw cap tube, allowed to cool to room temperature, and Abs_570 nm_ was measured in a spectrophotometer. A standard curve was prepared using glycine to determine amino acid concentrations in the samples. Percentage of proteolysis was calculated as amino acid concentration relative to the Proteinase K control without clovamide or cacao protein. The experiment was performed twice with five replicates each time. ANOVA and *post-hoc* Tukey-HSD were performed to determine statistical significance. Experiment was treated as a block in ANOVA.

### Pectolysis Assay

Pectolysis assays were adapted from methods by Torres et al. (2011) for detection of endo-polygalacturonase activity. Apple pectin (Sigma 93854) digestion by pectinase from *A. niger* (Sigma 17389) was used as a model reaction. Epigallocatechin gallate (EGCG, Sigma E4143), a known inhibitor of pectin methylesterases, was used as a positive control (Lewis et al., 2008; Jiang et al., 2014). Pectin precipitation by ruthenium red (Cayman Chemical 14339) was used for pectin quantification. Pectin solution (0.15% w/v) was prepared in 10 mM KOH and pectinase solution (0.025 mg/ml) was prepared in acetate buffer (0.1 M, pH 4.25). Each reaction consisted of 46 μl pectin solution, 50 μl pectinase solution or acetate buffer, 2 μl DMSO or 100 mM clovamide or 100 mM EGCG (final concentration 2 mM), and 2 μl of cacao protein (150 ng/μl) or protein storage buffer as described above. Assays to determine the pectinase enhancing effect of cacao proteins were generally the same but used 48 μl pectin solution and no DMSO. Reactions were incubated 45 min at 25°C. After incubation, 50 μl of the reaction was mixed with 950 μl of ruthenium red solution (0.3 mg/ml in acetate buffer with 0.05% v/v β-mercaptoethanol), briefly vortexed, and centrifuged for 5 min 14,000 × *g* to precipitate pectin. The supernatant was measured with a spectrophotometer (Abs_535 nm_), and pectin concentration was determined by comparison to a standard curve generated with serial dilutions of pectin. The concentration of pectin in a control reaction without pectinase and the concentration of pectin remaining in each reactions’ supernatant were used to calculate the percentage of pectin degraded. For pectinase inhibition assays, values were normalized to reactions without inhibitor. The pectinase inhibition assay was performed twice with three replicates each time. ANOVA and *post-hoc* Tukey-HSD were performed to determine statistical significance. Experiment was treated as a block in ANOVA.

### *Phytophthora* spp. Growth Inhibition Assays

All *Phytophthora* cultures were kindly provided by Dr. Brian Bailey (USDA-ARS, Beltsville, MD). *P. megakarya* isolate Ca-ZTH0145, *P. palmivora* isolate Gh-ER1349, and *P. tropicalis* isolate Eq-73-73 were growth on 20% V8-agar media (Jeffers and Martin, 1986). As previously described (Fister et al., 2016), mycelium plugs from the growing edge of the colony were collected 2 days after culture initiation and used to inoculate plates for growth inhibition assays.

Assay plates (60 × 15 mm) each contained 6 ml of media with either 2% (v/v) DMSO or 100 mM clovamide stock in DMSO (final concentration 2 mM). Two media were used for assays: V8-agar or Henniger/Casein/Pectin (“HenCasPec”) adapted from (Henniger, 1963). HenCasPec media consisted of (per liter): 0.4 g KH_2_PO_4_, 0.1 g CaCl_2_ (anhydrous), 0.1 g MgCO_3_, 0.02 g FeSO_4_•7H_2_O, 0.2 g succinic acid, 10 g glucose, 5 g sucrose, 1 mg thiamine•HCl, 30 mg beta-sitosterol, 8 g casein (Sigma C7078), 1 g pectin from apple (Sigma 93854), and 10 g agarose. The pH was adjusted to 7.0 with KOH and media was autoclaved. Cultures were incubated for 68 h and photographed. White mycelium was not easily visible on the white HenCasPec media so those plates were stained with 0.01% calcofluor (as Fluorescence Brightener 28, Sigma F3543) for 2 min and washed once with 1 M NaOH before photographing under 365 nm UV excitation. Colony areas were determined in ImageJ, and percent growth inhibition was calculated relative to the mean area of control plates for each experiment. Each experiment had four replicate plates and was performed twice with the exception of *P. palmivora* on HenCasPec media, which represents one experiment. ANOVA and *post-hoc* Tukey-HSD were performed to determine statistical significance. Experiment was treated as a block in ANOVA.

### Untargeted Metabolomics of Fruit Peel Tissue: Liquid Chromatography – Mass Spectrometry

Fruits/pods (4–5 month old) were surface sterilized by submerging with 100% ethanol for 20 s and allowed to dry in a sterile hood. A cork borer (3 mm) was used to make a small hole in the pod ~5 mm deep. Inside the holes, either a sterile V8-agar media plug (“mock”) or a plug with *P. palmivora* “GhER1349” mycelium (“Ppal”) was inserted. Pods were incubated at 27°C with a 16 h/8 h light/dark cycle in a plastic bag containing a sterile, wet paper towel to maintain humidity. At 72 h after inoculation, a cork borer (3 cm) was used to delineate a circle around the original infection site. The agar plug was removed and a potato peeler was used to collect the disk of tissue around the site of infection to a depth of ~0.25 cm (exocarp and ~2 mm mesocarp). Three such disks of tissue were collected per pod and pooled to make one replicate. Three replicates were collected for each treatment. For the “ICS1 mock” treatment, only two pods were available. To make a third replicate, tissue disks from pods 1 and 2 were collected and pooled. Tissue was flash frozen in liquid nitrogen and extracted and analyzed by LC-MS/MS as described above for leaf.

The experiment was repeated to determine the effect of wounding. Three pods of each genotype were wounded as above with a cork borer and three pods were left untreated. Tissue was collected after a 72-h incubation (as above). Samples were prepared for LC-MS/MS as above, except a 20 μl:1 mg solvent:tissue ratio was used for metabolite extraction in this experiment.

### Statistics

All LC-MS data were processed in XCMS Online (Tautenhahn et al., 2012) using Welch’s *t*-test for metabolite abundance comparisons between groups. Student’s *t*-tests, ANOVA, and *post-hoc* Tukey-HSD for mean separation were performed in JMP® Pro 14 (SAS Institute Inc., Cary, NC, 1989–2019) or GraphPad Prism 8. For duplicated experiments (proteolysis, pectolysis, *Phytophthora* spp. growth inhibition), experimental replicate was treated as a block in ANOVA to ensure there was no effect of experiment before data were pooled from both experiments.

## Data Availability Statement

Original LC-MS/MS datasets used in this study have been deposited in the MetaboLights database (Haug et al., 2020) under study ID MTBLS2154 and can be found here: https://www.ebi.ac.uk/metabolights/MTBLS2154.

## Author Contributions

BK, SM, and MG designed the research plan. G-XL, JL, and BK synthesized clovamide. BK performed all other experiments except for LC-MS/MS, which was done at the Pennsylvania State University Metabolomics Facility. SM, MG, and JL supervised experiments. BK analyzed data and drafted the manuscript. All authors contributed to the article and approved the submitted version.

### Conflict of Interest

The authors declare that the research was conducted in the absence of any commercial or financial relationships that could be construed as a potential conflict of interest.

## Supplementary Material

Supplementary Data File 1 MS/MS spectra for “Top 30 Metabolite Features”. Metabolite features “S-1” through “S-30” correspond to those presented in Figure 1 and Table 1. Annotations in bold represent putative molecular ions ([M-H]^−^).

Supplementary Data File 2 Supporting data for predicted annotations of HCAAs shown in Figure 2, and MS signal abundance in ‘ICS1’ and ‘Sca6’ extracts for each metabolite feature. Sheet 1 provides predicted fragments for putative HCAAs that are presented in Figure 2. Sheet 2 provides MS/MS spectra for the predicted HCAAs.

Supplementary Data File 3 MS/MS spectra for metabolite features indicated in PCA loading chart (Figure 7 in main text).
