# Supplementary material for: Clovamide, a Hydroxycinnamic Acid Amide, Is a Resistance Factor Against Phytophthora spp. in Theobroma cacao
Source: Front Plant Sci. 2020 Dec 23;11:617520. doi: 10.3389/fpls.2020.617520 (PMC7786005; doi:10.3389/fpls.2020.617520)
Supplement: Supplementary file 4 [file Data_Sheet_4.pdf]

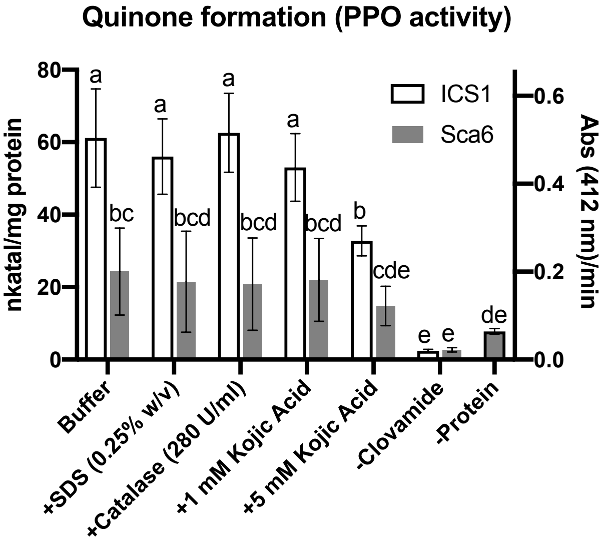

Supplemental Figure 1. Polyphenol oxidase (PPO) activity assay of stage C leaf protein extracts with controls. “Buffer” is control (McIlvaine’s buffer, pH 7). “SDS” = sodium dodecyl sulfate. “-Clovamide” represents protein only with no substrate. “-Protein” represent clovamide only with no added protein. One-way ANOVA was performed (p < 0.001) and pairwise student’s t-test were used for multiple comparisons. Groups with the same letter are not significantly different (p < 0.1). Error bars represent standard deviation.

A

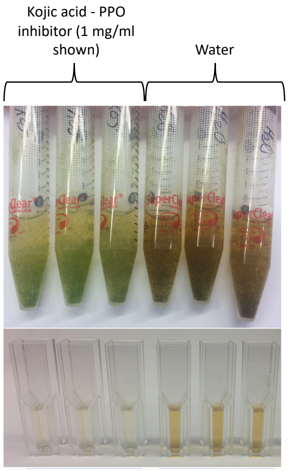

B

Perform absorbance scan and find absorbance maxima associated with PPO-mediated browning:
Leaf ground in water – ground in 5mM kojic acid

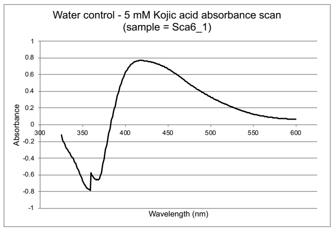

Peak wavelength: **418.3 ± 3.0 nm**
(3x ICS1 leaves and 3x Sca6 leaves)

Conclusion: use 418 nm absorbance as indicator of PPO-mediated browning after tissue grinding and incubation.

C

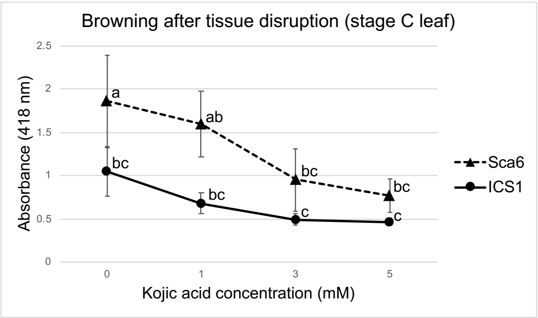

Kojic acid reduces browning in ground tissue.
Differences in browning can be observed between genotypes.
One-way ANOVA was performed ($p < 0.001$). Means are separated by Tukey-HSD. Treatments with the same letter are not significantly different ($p < 0.05$). Error bars represent SD.

Supplemental Figure 2. Development of browning assay used for Figure 4C,D. **(A)** Example of supernatant browning of ground 'Sca6' stage C leaf with and without kojic acid (PPO inhibitor). **(B)** Example absorbance scan (325-600 nm) of stage C leaf disc ground in water minus stage C leaf disc ground in 5 mM kojic acid solution. Absorbance maximum (~418 nm) indicates PPO-mediated browning. **(C)** Demonstration that kojic acid reduces browning measurement (Abs_{418nm}) in concentration-dependent manner and that 'Sca6' leaf discs produce more browning that 'ICS1'.

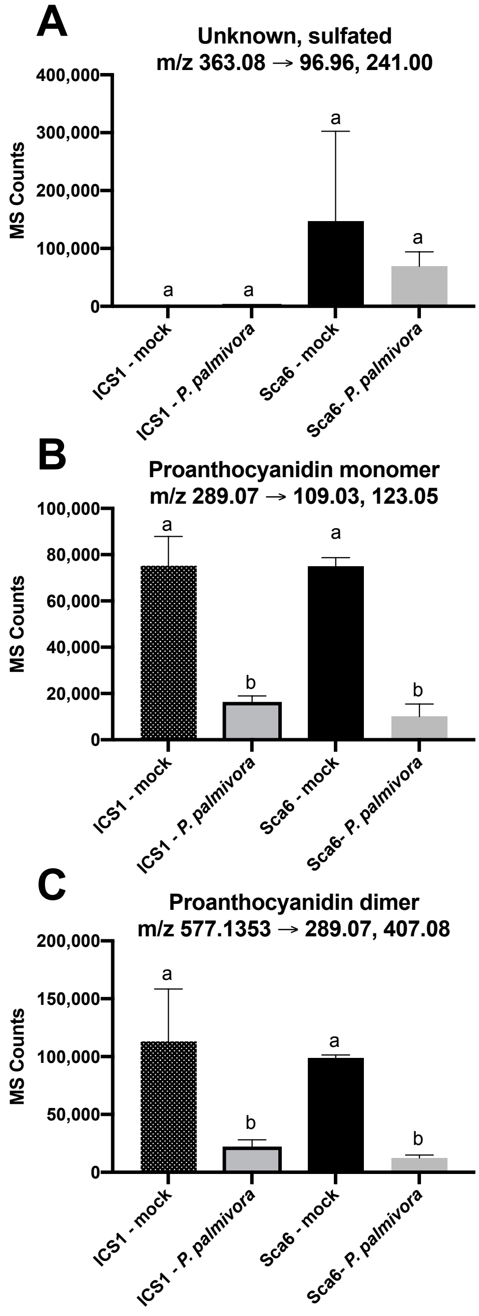

Supplemental Figure 3. Other metabolite features from LC-MS metabolomics of *P. palmivora* infection of pod that were indicated in the Loadings Chart (*Fig. 7B*). **(A)** Unknown sulfated metabolite. **(B)** A proanthocyanidin monomer (catechin or epicatechin). **(C)** A proanthocyanidin dimer (B type). MS Counts represent mass spectrometer signal intensity of peaks integrated in XCMS Online (Tautenhahn et al., 2012). Shared letters mean no difference by Tukey-HSD (p < 0.05, n = 3). Parent ion m/z values are suspected molecular ions ($[M-H]^-$) in **(B)** and **(C)**. Error bars represent standard deviation.

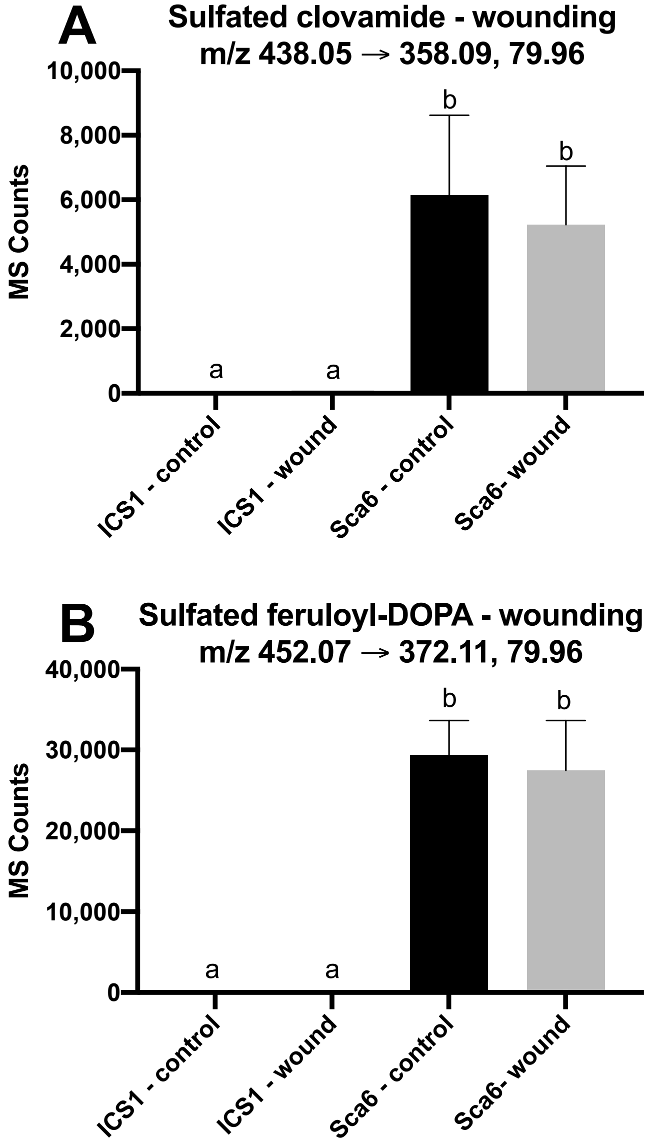

Supplemental Figure 4: Effect of wounding on two sulfated HCAAs in fruit/pod peel. **(A)** MS Signal of clovamide, arylsulfate in 'ICS1' and 'Sca6' control and wounded pods. **(B)** MS Signal of Feruloyl-DOPA, arylsulfate in 'ICS1' and 'Sca6' control and wounded pods. No induction of either compound by wounding was observed. Shared letters mean no difference by Tukey-HSD (*p* < 0.05, *n* = 3). Parent ion m/z values are suspected molecular ions ($[M\text{-}H]^-$). Error bars represent standard deviation.

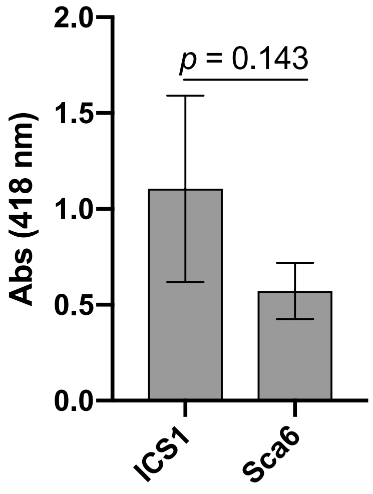

Supplemental Figure 5: Supernatant browning (Abs_{418nm}) from grinding ‘ICS1’ or ‘Sca6’ pod tissue in water. T-test, *n* = 3. Error bars represent standard deviation.

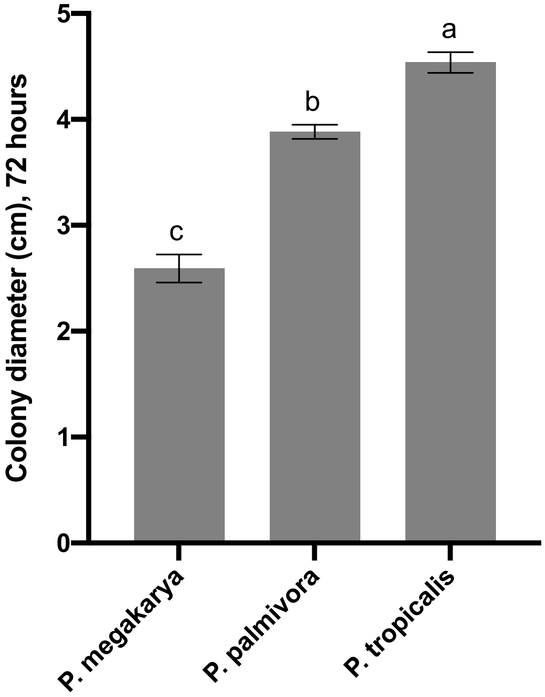

Supplemental Figure 6. Colony diameter of *P. megakarya, P. palmivora, P. tropicalis* in V8 media after 72 hours of growth. Shared letters mean no difference by Tukey-HSD ($p < 0.01$, $n = 8$). Error bars represent standard deviation.

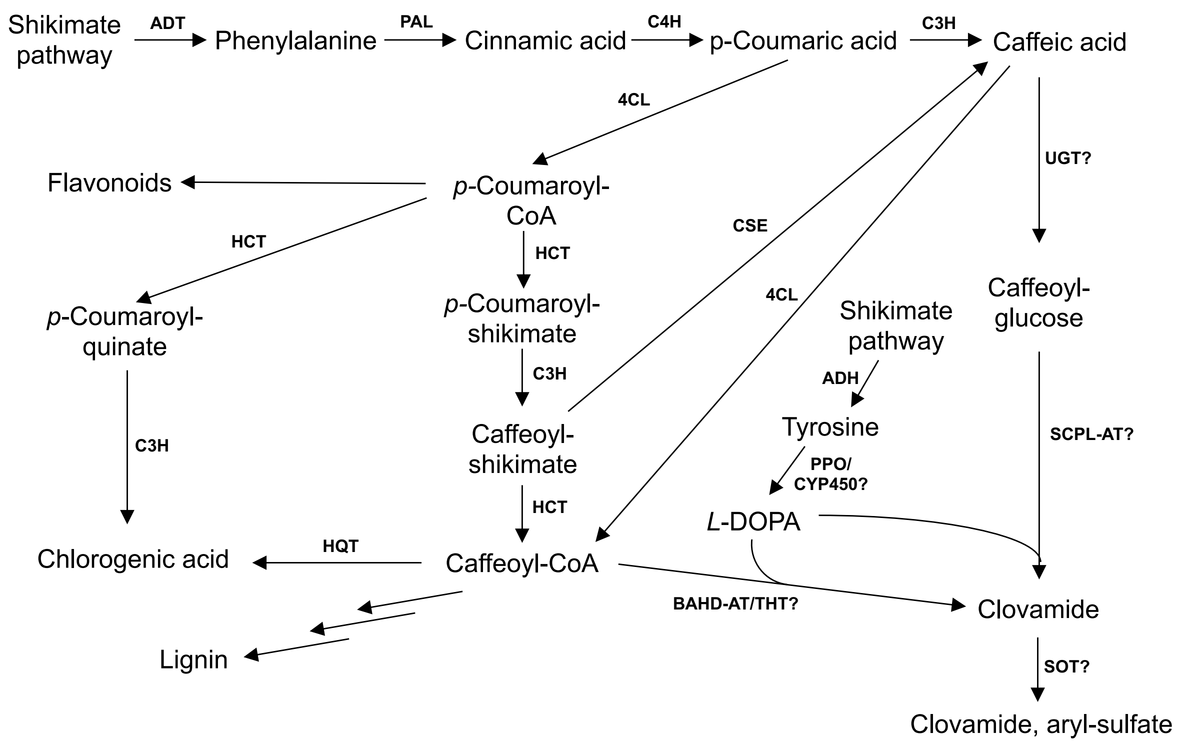

Supplemental Figure 7. Hypothetical biosynthetic pathways to clovamide from precursors derived from the Shikimate pathway. ADT = arogenate dehydratase, PAL = phenylalanine ammonia lyase, C4H = cinnamic acid 4-hydroxylase, C3H = *p*-coumaroyl 3' hydroxylase, 4CL = 4-coumaroyl CoA ligase, HCT = hydroxycinnamoyl-CoA shikimate/quinate hydroxycinnamoyl transferase, HQT = hydroxycinnamoyl-CoA quinate transferase, CSE = caffeoyl shikimate esterase, ADH = arogenate dehydrogenase, PPO = polyphenol oxidase, CYP450 = cytochrome p450 (tyrosine hydroxylase), *L*-DOPA = *L*-3, 4-dihydroxyphenylalanine, UGT = UDP-glucose dependent glycosyltransferase, SCPL-AT = serine carboxypeptidase-like acyltransferase, THT = hydroxycinnamoyl-CoA:tyramine-N-hydroxycinnamoyl transferase, BAHD-AT = BAHD acyltransferase – BAHD is acronym for first four substrates identified for this class - see Petersen (2016), SOT = sulfotransferase. Figure is based on: (Araji et al., 2014; Bouchez et al., 2019; Hirschmann et al., 2014; Knollenberg et al., 2018; Payyavula et al., 2015; Petersen, 2016; Polturak et al., 2016; Qian et al., 2019; Vanholme et al., 2013). Clovamide biosynthesis is known to occur from caffeoyl-CoA and L-DOPA via BAHD-AT in *Trifolium pratense* (Bouchez et al., 2019; Sullivan and Bonawitz, 2018), however the THT and UGT/SCPL-AT pathways presented are purely hypothetical and are provided as alternative hypotheses. Hypothetical pathways are based on similar acylations by hydroxycinnamoyl groups reported in the literature (D'Auria, 2006; Petersen, 2016). *L*-DOPA formation from tyrosine is catalyzed by a CYP450 in *Beta vulgaris* (Polturak et al., 2016) and likely by PPO in *Juglans regia* (Araji et al., 2014), although the responsible enzyme in cacao is currently unknown.

References

Araji, S., Grammer, T.A., Gertzen, R., Anderson, S.D., Mikulic-Petkovsek, M., Veberic, R., Phu, M.L., Solar, A., Leslie, C.A., Dandekar, A.M., *et al.* (2014). Novel roles for the polyphenol oxidase enzyme in secondary metabolism and the regulation of cell death in walnut. Plant physiology *164*, 1191-1203.

Bouchez, P., Teixeira Benites, V., Baidoo, E.E.K., Mortimer, J.C., Sullivan, M.L., Scheller, H.V., and Eudes, A. (2019). Production of clovamide and its analogues in Saccharomyces cerevisiae and Lactococcus lactis. Letters in Applied Microbiology *69*, 181-189.

D'Auria, J.C. (2006). Acyltransferases in plants: a good time to be BAHD. Curr Opin Plant Biol *9*, 331-340.

Hirschmann, F., Krause, F., and Papenbrock, J. (2014). The multi-protein family of sulfotransferases in plants: composition, occurrence, substrate specificity, and functions. Frontiers in plant science *5*, 556.

Knollenberg, B.J., Liu, J., Yu, S., Lin, H., and Tian, L. (2018). Cloning and functional characterization of a p-coumaroyl quinate/shikimate 3'-hydroxylase from potato (Solanum tuberosum). Biochem Biophys Res Commun *496*, 462-467.

Payyavula, R.S., Shakya, R., Sengoda, V.G., Munyaneza, J.E., Swamy, P., and Navarre, D.A. (2015). Synthesis and regulation of chlorogenic acid in potato: Rerouting phenylpropanoid flux in HQT-silenced lines. Plant biotechnology journal *13*, 551-564.

Petersen, M. (2016). Hydroxycinnamoyltransferases in plant metabolism. Phytochemistry Reviews *15*, 699-727.

Polturak, G., Breitel, D., Grossman, N., Sarrion-Perdigones, A., Weithorn, E., Pliner, M., Orzaez, D., Granell, A., Rogachev, I., and Aharoni, A. (2016). Elucidation of the first committed step in betalain biosynthesis enables the heterologous engineering of betalain pigments in plants. The New phytologist *210*, 269-283.

Qian, Y., Lynch, J.H., Guo, L., Rhodes, D., Morgan, J.A., and Dudareva, N. (2019). Completion of the cytosolic post-chorismate phenylalanine biosynthetic pathway in plants. Nature Communications *10*, 15.

Sullivan, M.L., and Bonawitz, N.D. (2018). Spectrophotometric determination of reaction rates and kinetic parameters of a BAHD acyltransferase using DTNB (5, 5′-dithio-bis-[2-nitrobenzoic acid]). Plant science *269*, 148-152.

Vanholme, R., Cesarino, I., Rataj, K., Xiao, Y., Sundin, L., Goeminne, G., Kim, H., Cross, J., Morreel, K., Araujo, P., *et al.* (2013). Caffeoyl Shikimate Esterase (CSE) Is an Enzyme in the Lignin Biosynthetic Pathway in Arabidopsis. Science (New York, NY) *341*, 1103.
